# An item response theory analysis of the matrix reasoning item bank (MaRs-IB)

**DOI:** 10.3758/s13428-023-02067-8

**Published:** 2023-04-05

**Authors:** Samuel Zorowitz, Gabriele Chierchia, Sarah-Jayne Blakemore, Nathaniel D. Daw

**Affiliations:** 1 Princeton Neuroscience Institute, Princeton University, Princeton, NJ USA; 2 Department of Psychology, University of Cambridge, Downing Street, Cambridge, UK; 3 Department of Psychology, Princeton University, Princeton, NJ USA

**Keywords:** Item response theory, Matrix reasoning, Progressive matrices, Speed-accuracy trade-off

## Abstract

Matrix reasoning tasks are among the most widely used measures of cognitive ability in the behavioral sciences, but the lack of matrix reasoning tests in the public domain complicates their use. Here, we present an extensive investigation and psychometric validation of the matrix reasoning item bank (MaRs-IB), an open-access set of matrix reasoning items. In a first study, we calibrate the psychometric functioning of the items in the MaRs-IB in a large sample of adult participants (*N* = 1501). Using additive multilevel item structure models, we establish that the MaRs-IB has many desirable psychometric properties: its items span a wide range of difficulty, possess medium-to-large levels of discrimination, and exhibit robust associations between item complexity and difficulty. However, we also find that item clones are not always psychometrically equivalent and cannot be assumed to be exchangeable. In a second study, we demonstrate how experimenters can use the estimated item parameters to design new matrix reasoning tests using optimal item assembly. Specifically, we design and validate two new sets of test forms in an independent sample of adults (*N* = 600). We find these new tests possess good reliability and convergent validity with an established measure of matrix reasoning. We hope that the materials and results made available here will encourage experimenters to use the MaRs-IB in their research.

**Supplementary Information:**

The online version contains supplementary material available at 10.3758/s13428-023-02067-8.

## Introduction

Matrix reasoning tasks are among the most widely used measures of cognitive ability in the behavioral sciences. Much of their popularity undoubtedly reflects their versatility of use. Matrix reasoning tasks are strong (albeit impure) indicators of general intelligence (Gignac, 2015) and working memory capacity (Kane et al., 2004; Unsworth & Engle, 2005). They are predictive of important real-world outcomes such as childhood academic achievement (Roth et al., 2015) and performance on college entrance exams (Frey & Detterman, 2004; Koenig, Frey, & Detterman, 2008). In low-stakes testing settings (i.e., where participants incur little or no cost for poor performance), matrix reasoning tasks can additionally function as measures of motivation, willingness to expend mental effort, and other facets of personality (Duckworth, Quinn, Lynam, Loeber, & Stouthamer-Loeber, 2011; Gignac, Bartulovich, & Salleo, 2019). In studies of psychiatric populations, performance on matrix reasoning tasks have also been used to control for general disruptions to cognitive ability when specific domains of cognition are of primary interest (Gillan, Kosinski, Whelan, Phelps, & Daw, 2016; Rouault, Seow, Gillan, & Fleming, 2018; Moutoussis et al., 2021).

Unfortunately, there are a number of obstacles to using matrix reasoning tasks as part of behavioral research. One challenge is the problem of copyright. Many of the most prominent matrix reasoning tasks, such as the WAIS and WASI matrix reasoning subtests (Wechsler, 1999; 2008), are not free to use and have legal restrictions against digitization. A second challenge is that across all of the matrix reasoning tests in the public domain, there are relatively few unique items available. The Hagen matrices test (Heydasch, 2014) and ICAR matrix reasoning test (Condon & Revelle, 2014), for example, have only 20 and 16 items, respectively. The availability of only a limited number of items raises the possibility of repeat exposure effects, which threaten the validity of these measures (Ng, 1974; Bors & Vigneau, 2003). This problem is exacerbated in the current era of online experiments, where multiple groups of researchers may be inadvertently administering the same test to the same participants recruited from the same online labor platforms.

**Fig. 1:**
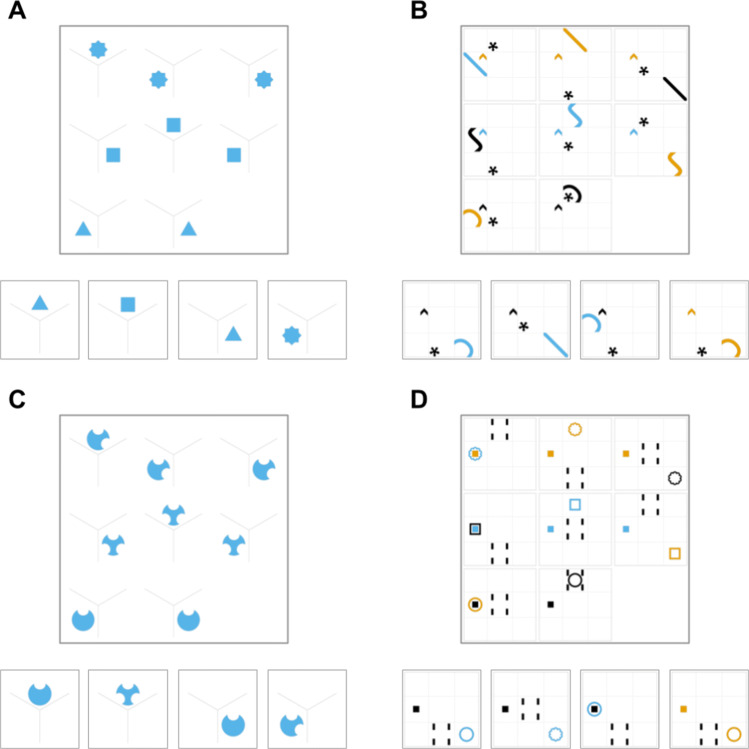
Example items from the MaRs-IB. (A) A simple item containing one element per cell and two rules (i.e., a change of shape and position). (B) A harder item containing three elements and six rules (i.e., three position changes, two color changes, one shape change). (C)/(D) Alternate versions of Items A and B, respectively, matched on complexity (i.e., number of elements and rules). In all panels, the first option is the correct response

Noting these challenges, Knoll, Fuhrmann, Sakhardande, Stamp, Speekenbrink & Blakemore (2016) / Chierchia, Fuhrmann, Knoll, Pi-Sunyer, Sakhardande & Blakemore, (2019) developed and made publicly available the matrix reasoning item bank (MaRs-IB), a collection of 80 matrix reasoning puzzles. Each puzzle in the MaRs-IB consists of a 3x3 matrix containing geometric shapes in eight out of nine cells (Fig. 1). Across cells, some or all of these shapes change according to a number of abstract rules. Based on these patterns, participants must deduce which of four response options correctly completes the matrix. The puzzles in the MaRs-IB vary in their complexity, both in the number of elements per cell and number of rules determining the relations of these elements across cells (Fig. 1a, b). Further increasing the reusability of the MaRs-IB, each puzzle (hereafter referred to as an item template) has six clones. An item’s clones are equally complex (i.e., possess the same number of elements and rules) but may vary in their distractors (two unique sets per template) or perceptual features (three unique shape sets per template; Fig. 1c, d). Thus, the MaRs-IB addresses the copyright and limited reuse issues associated with other matrix reasoning tests.

Chierchia et al., (2019) conducted an initial study of the psychometric properties of the MaRs-IB in which a sample of 659 adult, adolescent, and child participants had 8 min to complete as many MaRs-IB items as possible. Of note, items were presented in the same order to all participants. The authors found that measures of performance on this task had good split-half and test–retest reliability. Moreover, they found that participants’ ability to solve MaRs-IB puzzles was moderately predictive of their performance on a working memory task and the ICAR matrix reasoning test, indicating satisfactory convergent validity. At the conclusion of this study, Chierchia et al., (2019) made publicly available a summary of the difficulty (i.e., proportion correct responses) of every item template and clone in the MaRs-IB so that other researchers could construct their own test measures of custom difficulty and duration.

Two important limitations of the experiment conducted by Chierchia et al., (2019) undermine the utility of these summary statistics and, consequently, impede the design of new matrix reasoning tests using the MaRs-IB. The first is that, given the fixed-order experiment design, there were relatively few responses collected for the majority of the items. Indeed, only 42 of 80 item templates were completed by 100 or more participants. As such, one cannot be confident about the true difficulty of a majority of the items (an issue that is exacerbated when considering that each item template has six clones). A second issue is participants were able to choose for themselves whether to prioritize accuracy (number of correct responses) or productivity (number of items reached). As a consequence, response data for items that came later in the fixed-order task are increasingly likely to have been produced by participants sacrificing accuracy for speed, thereby biasing the proportion correct measure for these items (see the Supplementary Materials for evidence of this effect). Therefore, additional investigation of the functioning and psychometric properties of the MaRs-IB is warranted.

Here, we provide a more extensive study and psychometric validation of the MaRs-IB. The most important contribution of the current work is the characterization of (almost) every item in the MaRs-IB using item response theory (IRT; Embretson & Reise, 2013; De Ayala, 2013). IRT models confer several important advantages for the purposes of psychometric validation. First, they provide an estimate of the *difficulty* and *discrimination* of each item, where the latter is an index of the reliability of an item. Using these quantities, IRT models in turn allow experimenters to compute the test information function (TIF), which quantifies the reliability of a test for measuring performance given a level of ability. Finally, IRT models make possible optimal test assembly (Van der Linden, 1998), or the design of new tests with maximal reliability given researcher-specified constraints (e.g., test duration or difficulty).

A second contribution provided here is an analysis of how item properties shape the psychometric functioning (i.e., difficulty, discrimination) of items in the MaRs-IB. Using explanatory item response modeling (De Boeck & Wilson, 2004; Wilson, De Boeck, & Carstensen, 2008), we specifically investigate how item complexity, i.e., the number of elements and number of rules, determine item functioning. We studied these two item attributes because they have been previously identified as among the strongest predictors of item difficulty in matrix reasoning tasks (Embretson, 1998; Primi, 2001). This explanatory analysis provides another means by which to validate the design of the MaRs-IB; that is, to determine whether the MaRs-IB exhibits the same associations between item properties and functioning as found in other established matrix reasoning tests. As a secondary benefit, explanatory IRT models yield more precise estimates of item parameters (Neuhaus & McCulloch, 2006), which in turn help to ensure that any new MaRs-IB tests designed based on those estimates function as expected.

A third contribution is an investigation of the exchangeability of item clones in the MaRs-IB, or if clones are (approximately) psychometrically equivalent. Establishing whether the MaRs-IB possesses this property is critical for the design of new matrix reasoning tasks using these items. If item clones are exchangeable, then new parallel test forms of equivalent difficulty and reliability can be generated simply by substituting an item clone from one shape set for another. To test for exchangeability, we use additive multilevel item structure (AMIS) models (Geerlings, Glas, & Van Der Linden, 2011; Cho, De Boeck, Embretson, & Rabe-Hesketh, 2014; Lathrop & Cheng, 2017). These models enable us to quantify the variability in item difficulty and discrimination across clones, by distractor type and shape set, which must be negligible if clones are to be treated as exchangeable.

The remainder of the paper proceeds as follows. First, we report a calibration study in which a large sample of adult participants completed a number of items from the MaRs-IB. Using their response data, we fitted a series of multilevel item structure models in order to estimate item parameters for each item (and their clones). Using these same models, we also interrogate the relationship between item attributes and item functioning. We conclude by reporting a second validation study, in which an independent sample of participants (*N* = 600) completed one of five novel MaRs-IB test forms. These tests—designed using optimal test assembly and the item parameter estimates from the first study—were found to have good psychometric properties and convergent validity with an established measure of matrix reasoning ability. This second study provides a blueprint for how researchers can construct new MaRs-IB test measures based on the results of our first study.

All data, code, model outputs (including the estimated item parameters), and tutorials are publicly available at: https://github.com/ndawlab/mars-irt. The MaRs-IB stimuli are publicly available at: https://osf.io/g96f4/.

## Calibration study

### Objectives

The purpose of the first study was to calibrate the items in the MaRs-IB using response data collected from a large number of adult participants from the general population. In particular, we sought to accomplish the following three aims: (1) to quantify the psychometric properties (i.e., difficulty, discrimination) of the items in the MaRs-IB using item response models; (2) to measure the associations between item complexity and functioning; and (3) to determine whether item clones in the MaRs-IB are psychometrically equivalent and exchangeable.

### Methods

#### Participants

A total of *N* = 1584 participants were recruited from the Prolific Academic platform (https://www.prolific.co) to participate in an online experiment between July and August, 2021. Participants were eligible if they were 18 years or older and lived in the United States. Total study duration was approximately 6.4 min (sd = 2.4) per participant. Participants received monetary compensation for their time (rate: $10 USD/hr), plus a performance-based bonus up to $0.50 USD. On average, participants earned a total of $1.30 USD (sd = $0.10). We offered performance bonuses as they have been found to motivate performance in low-stakes testing environments (Duckworth et al., 2011; Gignac, 2018). This study was approved by the Institutional Review Board of Princeton University (#7392), and all participants provided informed consent.

To ensure data quality, the data from multiple participants were excluded prior to analysis (see Exclusion Criteria below) leaving the data from *N* = 1501 participants for analysis. In these participants, the majority identified as women (men: *N* = 670; women: *N* = 811; non-binary or other: *N* = 13; rather not say: *N* = 2) and the average age was 28.7 years old (sd = 9.9; range, 18–74). The sample was relatively well educated with the majority having completed a bachelor’s degree (*N* = 507) or master’s degree or higher (*N* = 322). Comparatively fewer participants completed only some college (*N* = 471), only a high school degree (*N* = 199), or preferred not to say (*N* = 2).

#### Procedure

After providing consent, participants completed 16 items from the MaRs-IB. The design of the MaRs-IB items have been described previously (Chierchia et al., 2019). Briefly, each MaRs-IB item consists of a 3x3 matrix. Eight of the nine cells contain abstract shapes, while one cell on the bottom right-hand side of the matrix is empty. Participants were instructed to “complete the matrix” by identifying the missing shape from among four possible alternatives.

The presentation of each item was preceded by a fixation cross, which lasted for 1200 ms. Upon presentation of the item, participants were given up to 30 s to solve the puzzle. After 25 s elapsed, a clock appeared to count down the remaining 5 s. A trial ended when participants responded, or after 30 s had elapsed without response. Before the trials began, participants reviewed instructions and were made to correctly complete three practice items. Participants were instructed to respond carefully, but to guess if they could not solve the puzzle before the timer ran out. The format of the experiment and instructions were adapted from code publicly released as part of the original study (https://app.gorilla.sc/openmaterials/36164). The task was programmed in jsPsych (De Leeuw, 2015) and distributed to participants via the web using custom software (available at https://github.com/nivlab/nivturk).

In order to ensure sufficient sampling of every item template and clone in the MaRs-IB, participants were administered a pseudorandomly-selected set of 16 out of 64 total items.^1^ Item sets were constructed as follows: We subdivided the 64 items into 16 sets of four based on their dimensionality (a measure of item complexity, defined in Chierchia et al., 2019). Participants were randomly assigned one item from each of the 16 sets. As such, all participants received test forms of roughly equal difficulty.

Importantly we also counterbalanced the assignment of item clones across participants, such that we had an approximately uniform number of responses available for each clone by shape set (1, 2, or 3) and distractor type (minimal difference, MD, or paired difference, PD). Distractor type refers to the two strategies used to generate the distractor response options in the MaRs-IB. The MD strategy produces distractors that are variations of the target response. The MD strategy has the advantage of preventing pop-out effects (where the correct response “pops out” among the possible responses), but has the disadvantage of theoretically allowing participants to solve an item by looking at the response options only. In contrast, the PD strategy produces distractors that are have at least one feature in common with the target response. The PD strategy prevents participants from solving items by looking at the response options alone, but can potentially induce pop-out effects. We note that, for complex items with many elements, it is not possible to prevent both at the same time.

#### Exclusion criteria

The data from multiple participants who completed the experiment were excluded prior to analysis for one or more of the following reasons: rapid guessing (responding in less than 3 s)^2^ on four or more trials (*N* = 70); failing to respond on four or more trials (*N* = 8); or minimizing the browser window to a dimension smaller than the minimal requirements (*N* = 7). In total, 83 of 1584 (5.2%) participants were excluded leaving the data from *N* = 1501 participants available for analysis. Across these participants, each item (64 total) was administered approximately 375 times (sd = 12) and each item clone (384 total) was administered approximately 62 times (sd = 7).

#### Response time analysis

We investigated the relationship between accuracy and response time using a mixed effects (random intercepts) linear regression model. Trial-wise (log-transformed) response times were predicted as a function of trial accuracy, rest score (participants’ observed scores on all other items), and item difficulty (one minus the proportion correct for that item). The mixed-effects model was estimated using the *statsmodels* python package (v0.12.2; Seabold & Perktold, 2010).

#### Item response models

We employed item response models to characterize the psychometric properties of each item. Specifically, we used additive multilevel item structure (AMIS) models (Geerlings et al., 2011; Cho et al., 2014; Lathrop & Cheng, 2017). In AMIS models, item parameters are defined according to a hierarchical structure in which item clones are nested in item templates. The foundation of all the models used here is the three-parameter logistic (3PL) item response model, where the probability of a correct response (*y*_*ijk*_ = 1) for person *i* on item clone *k* belonging to item template *j* is:

1 $$  p(y_{ijk} = 1) = \gamma_{jk} + (1-\gamma_{jk}) \cdot \text{logit}^{-1} \left( \alpha_{jk} \cdot \theta_{i} - \beta_{jk} \right) $$

where *𝜃*_*i*_ is the latent ability for person *i*, and *β*_*jk*_, *α*_*jk*_, and *γ*_*jk*_ are the difficulty, discrimination, and guessing parameters for item clone *k* of item family *j*. Because estimates of guessing parameters are often unreliable in the absence of very large amounts of response data (Han, 2012), we fixed the guessing parameter for every item to the nominal guessing rate (*γ*_*jk*_ = 0.25).

The difficulty and discrimination parameters were estimated following an additive multilevel item structure. Concretely, the difficulty of item clone *k* belonging to item template *j* was expressed as:

2 $$ \beta_{jk} = \mu_{\beta} + {\sum\limits_{n=1}^{N}} Q_{jn} \delta_{\beta n} + \epsilon_{\beta j} + {\sum\limits_{m=1}^{M}} R_{km} \delta_{\beta m} + \epsilon_{\beta k} $$

To elaborate, item difficulty parameter was modeled as an intercept (*μ*_*β*_), reflecting the average difficulty across all items, and four additional components:

1. ${\sum }_{n=1}^{N} Q_{jn} \delta _{\beta n}$: the effect of item template (level 1) attributes (described below) on difficulty, where *Q*_*jn*_ is the value of attribute *n* for item template *j*, and *δ*_*βn*_ is the effect of attribute *n*. This component is the fixed effects contribution to item template difficulty.
2. *𝜖*_*βj*_: the template (level 1) residual, or the residual variability in difficulty of the item template unexplained by its attributes. This component is the random effects contribution to item template difficulty.
3. ${\sum }_{m=1}^{M} R_{km} \delta _{\beta m}$: the effect of item clone (level 2) attributes on difficulty, where *R*_*km*_ is the value of attribute *m* for item clone *k*, and *δ*_*βm*_ is the effect of attribute *m*. This component is the fixed effects contribution to item clone difficulty.
4. *𝜖*_*βk*_: the clone (level 2) residual, or residual variability in difficulty of the item clone unexplained by its attributes. This component is the random effects contribution to item clone difficulty.

So too, item discrimination parameters were expressed as the sum of an equivalent set of components:

3 $$ \alpha_{jk} = \mu_{\alpha} + {\sum\limits_{n=1}^{N}} Q_{jn} \delta_{\alpha n} + \epsilon_{\alpha j} + {\sum\limits_{m=1}^{M}} R_{km} \delta_{\alpha m} + \epsilon_{\alpha k} $$

where the interpretation of each component is the same as for item difficulty.

In our analyses, we considered two template (level 1) attributes: element number and rule number. These have previously been identified as key determinants of item difficulty in matrix reasoning tasks (Embretson, 1998; Primi, 2001). Element number refers to the number of geometric shapes in each cell of a matrix, whereas rule number refers to the number of relationships that govern the changes among these shapes across cells. In the MaRs-IB, there are four principal rule types: size change, color change, position change, and shape change. Across item templates, element number ranges from one to four (median = 2) and rule number ranges from one to six (median = 3). Element and rule number are uncorrelated across item templates (Spearman’s *ρ* = 0.176), and were determined for each item via manual annotation.

At the clone level (level 2), we modeled an additional two attributes: distractor type and mean response time. Distractor type refers to whether a clone uses MD-type versus PD-type distractors, whereas mean response time refers to the average time participants spent deliberating on that item clone. We note that, in contrast to the other modeled attributes, average response time is not an intrinsic property of an item. We elected to include it in our models, however, because including response time as a covariate has previously been shown to improve parameter estimation (Bertling & Weeks, 2018).^3^ To preclude any collinearity between the attributes, mean response time was orthogonalized with respect to the three other item attributes; as such, any effects of response time reflects residual structure after accounting for all other item attributes.

We deliberately chose not to incorporate shape set (i.e., 1, 2, or 3) as a clone-level attribute. The allocation of geometric shapes to item clones in the MaRs-IB is complex, such that not all clones of the same shape set share the same perceptual elements (see the Supplementary Materials for a full discussion). In other words, shape set does not constitute an interpretable categorical variable and was therefore not included in the model. Instead, the contribution of perceptual elements to item functioning is captured by the clone-level residual variability terms of the item structure models.

We fit a series of nested AMIS models in order to identify the model that best predicted participants’ response data. The models varied in how item parameters were specified, with each successive model allowing for greater flexibility in item parameter estimation. We fit the models in two waves. In the first, item discrimination was fixed (*α* = 1)^4^ in order to identify the best structure for the item difficulty independent of item discrimination. In the first wave, we fit three models:

- Model 1: Item difficulty is a function of item template attributes (level 1) and item clone attributes (level 2). In this model, item difficulty is predicted solely by item attributes (i.e., element number, rule number, distractor type, response time).
- Model 2: Item difficulty is a function of all item attributes (level 1/2) and level 1 residuals. The inclusion of the level 1 residual serves to quantify the magnitude of residual variability in item difficulty, across item templates, unexplained by the level 1 attributes (i.e., element number, rule number).
- Model 3: Item difficulty is a function of all item attributes (level 1/2) and level 1/2 residuals. The inclusion of the level 2 residual serves to quantify the magnitude of residual variability in item difficulty, across item clones, unexplained by the level 2 attributes (i.e., distractor type, mean response time).

In the second wave of model fitting, item discrimination was specified as a free parameter to be estimated. Moreover, in the second set of models, the structure of the item difficulty parameters were specified according to the best-fitting model from the first wave. In the second wave, we fit an additional three models:

- Model 4: Item discrimination is a function of item template attributes (level 1) and item clone attributes (level 2). In this model, item discrimination is predicted solely by item attributes (i.e., element number, rule number, distractor type, response time).
- Model 5: Item discrimination is a function of all item attributes (level 1/2) and level 1 residuals. The inclusion of the level 1 residual serves to quantify the magnitude of residual variability in item discrimination, across item templates, unexplained by the level 1 attributes (i.e., element number, rule number).
- Model 6: Item discrimination is a function of all item attributes (level 1/2) and level 1/2 residuals. The inclusion of the level 2 residual serves to quantify the magnitude of residual variability in item discrimination, across item clones, unexplained by the level 2 attributes (i.e., distractor type, mean response time).

The AMIS modeling framework provides a natural means of accomplishing the three objectives of the calibration study. Via all of the models above, we obtain estimates of the psychometric properties (i.e., difficulty, discrimination) of every item template and clone in the MaRs-IB (Aim 1). Through the level 1 fixed effects, we are able to test for associations between item complexity (i.e., element and rule number) and item functioning (Aim 2). We predicted that both attributes would be positively associated with item difficulty; that is, all else equal, items with either a greater number of elements and rules would be more difficult. In contrast, we had no hypotheses regarding the association between these attributes on item discrimination.

Finally, through both the level 2 fixed and random effects, we can determine whether item clones in the MaRs-IB are exchangeable (Aim 3). For example, a credible association between distractor type and either item difficulty or discrimination would entail that clones are not psychometrically equivalent. Moreover, if we found any level 2 residual variance term were substantial, that would similarly indicate that not all item clones are equivalent due to unmodeled attributes (e.g., perceptual features).

#### Person-level effects

In each of the six models described above, we incorporated person-level attributes as predictors of latent ability (*𝜃*_*i*_) following an explanatory item response modeling approach (Wilson et al., 2008):

4 $$  \theta_{i} = {\sum\limits_{p=1}^{P}} X_{ip} \rho_{p} + \epsilon_{i} $$

where *X*_*ip*_ is the value of attribute *p* for person *i*, *ρ*_*p*_ is the partial correlation of person attribute *p*, and *𝜖*_*i*_ is variance in ability unexplained by the person-level attributes.

We estimated person-level abilities as a function of four covariates: age, gender, mean response time, and Δ response time. This last term reflects the degree of change in a participant’s response time as a function of item difficulty (defined as one minus the proportion of correct responses for that item), and was included because of its relationship to participants’ performance (see Results). Mean response time and Δ response time were calculated for each participant simultaneously via linear regression, where a given participant’s (log-transformed) response times were regressed against an intercept and item difficulty. All covariates were standard-scored prior to analysis with the exception of gender, which was binary coded (male = -0.5, female = 0.5).

#### Model fitting

All models were estimated within a Bayesian framework using Hamiltonian Monte Carlo as implemented in Stan (v2.22; Carpenter et al., 2017). For all models, four separate chains with randomized start values each took 7500 samples from the posterior. The first 5000 samples from each chain were discarded. As such, 10,000 post-warmup samples from the joint posterior were retained. The $\hat {R}$ values for all parameters were less than 1.01, indicating acceptable convergence between chains, and there were no divergent transitions in any chain.

During estimation, the item discrimination parameters were restricted to be in the range *α*_*jk*_ ∈ [0,5]. To ensure the identifiability of all models, person abilities were constrained to have a mean of zero and a variance of 1.0. Specifically, the residual variance of latent ability was specified as $V(\epsilon ) = 1 - \sum {\rho _{p}^{2}}$. The specification of the model priors are detailed in the supplement.

#### Model comparison

The goodness of fit of the models was compared using Bayesian leave-one out cross-validation (Vehtari, Gelman, & Gabry, 2017), which has been found to perform better than more traditional information criteria for comparing item response models (Luo & Al-Harbi, 2017). We computed the conditional leave-one-cluster out (LOCO) cross-validation (Merkle, Furr, & Rabe-Hesketh, 2019), which measures a model’s ability to generalize to held-out items (rather than held-out responses or held-out participants)—i.e., for inferring generalization to additional items constructed using the same features rather than to additional participants sampled from the same population.

#### Goodness-of-fit

The fit of the best-fitting model to the data was evaluated using posterior predictive model checking (Gelman, Meng, & Stern, 1996; Levy & Mislevy, 2017). A sample of predicted responses was generated for each sample of simulated parameters and a posterior predictive *p* (PPP) value was computed based on two discrepancy statistics: a chi-square $\left (\chi ^{2}_{NC} \right )$ discrepancy measure based on the observed score distribution (Sinharay, Johnson, & Stern, 2006) and the standardized generalized dimensionality discrepancy measure (SGDDM; Levy, Xu, Yel, & Svetina, 2015).

The $\chi ^{2}_{NC}$ discrepancy statistic is a measure of item fit based on the comparison of observed and expected proportions of participants at each score level (we ignore scores ≤ 1, achieved by only seven participants). A global measure of fit at the test level is obtained by summing the discrepancy values over the groups. In turn, SGDDM measures the mean absolute conditional correlation between all pairs of items; that is, the SGDDM is an index of residual inter-item correlations unexplained by the model. The SGDDM is important in this context because it tests for local dependence across items, the presence of which might otherwise lower the efficiency of future test forms via the inclusion of nonindependent or redundant items. For both discrepancy statistics, the PPP value is the proportion of draws in which the posterior predictive discrepancy is equal to or higher than the realized discrepancy. A poor model fit to the data is indicated when the PPP values are extreme (PPP ≤ 0.05).

As a final validation of the best-fitting model, we also performed a parameter recovery analysis. In this analysis, we generated 100 artificial datasets with sampling and statistical properties matched to what we observed empirically. That is, we generated datasets with 1500 participants and 384 item clones (nested in 64 item templates), where the item parameters were randomly generated and matched to the observed distribution of item parameters. We then fit the best-fitting model to each artificial dataset, and quantified the consistency between the ground-truth and recovered model parameters. The complete details of this procedure can be found in the Supplementary Materials.

### Results

#### Descriptive statistics

**Fig. 2:**
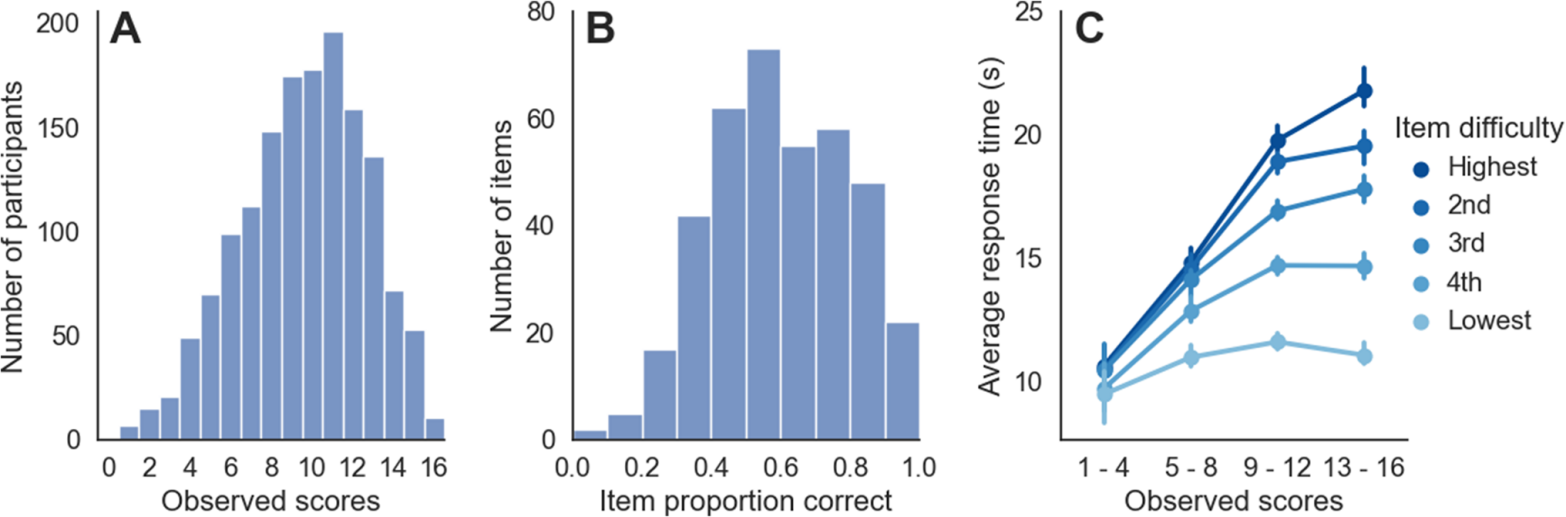
Summary of performance on the MaRs-IB items. (A) The distribution of total scores across all 1501 participants. (B) The distribution of proportion correct responses across all 384 items. (C) The distribution of participants’ median response times across items broken down by participants’ total scores and item difficulty (one minus proportion correct responses). *Error bars* indicate bootstrapped 95% confidence intervals around the mean

On average, participants completed 9.6 of 16 items correctly (sd = 3.1, IQR = 8.0–12.0; Fig. 2a). Timing out occurred on only 1.8% of trials (these were subsequently coded as incorrect responses). A total of 43 participants (2.9%) performed below chance (i.e., fewer than four items correct), while only 11 participants (0.7%) solved all 16 of their items. Thus, over 95% of observed scores were in a reasonable range. These results corroborate those reported by Chierchia et al., (2019) in that there were no obvious ceiling or floor effects in performance, and that the majority of participants were sufficiently motivated to participate in spite of the low-stakes testing environment.

Across all 384 item clones, the average proportion correct was 0.60 (sd = 0.19, IQR = 0.45–0.74; Fig. 2b). A total of 12 item clones (3.1%) exhibited performance levels beneath chance, and only 22 item clones (5.7%) had performance levels near ceiling (≥ 90%). Across items, the proportion of correct responses was negatively correlated with rule number (*ρ* = -0.482, *p* < 0.001). These results further corroborate those reported by Chierchia et al., (2019) in that most items in the MaRs-IB exhibited good functioning, with performance at ceiling or floor for only a few item clones.

#### Response time analysis

The results of the linear mixed-effects model of response times are summarized in Table 1. On average, participants spent 15.9 s (sd = 7.2 s) per item. There were significant main effects of accuracy (*β* = 0.067, *t* = 9.84, *p* < 0.001), rest score (*β* = 0.125, *t* = 16.95, *p* < 0.001), and item difficulty (*β* = 0.137,*t* = 44.33,*p* < 0.001). In other words, response times were slower on average for correct responses, better- performing participants, and more difficult items. The positive association between response time and accuracy is consistent with a speed–accuracy trade-off in participants’ performance. This interpretation is corroborated by the main effect of rest score, where better-performing participants were slower overall.

**Table 1:** Summary of the mixed effects linear regression model predicting log-transformed response time as a function of trial accuracy, participant rest score (total correct on all other items), and item difficulty (one minus the proportion correct for an item)

Predictor	Coef. (se)	Z-score (*p* value)	95% CI
Accuracy	0.039 (0.007)	5.367 (< 0.001)	0.025–0.053
Score	0.167 (0.009)	19.204 (< 0.001)	0.150–0.184
Difficulty	0.073 (0.005)	13.292 (< 0.001)	0.062–0.083
Accuracy x Score	-0.064 (0.007)	-8.764 (< 0.001)	-0.078−-0.050
Accuracy x Difficulty	0.097 (0.007)	14.351 (< 0.001)	0.084–0.110
Score x Difficulty	0.032 (0.005)	6.549 (< 0.001)	0.023–0.042
Accuracy x Score x Difficulty	0.012 (0.006)	1.955 (0.051)	-0.000–0.025

lme4 syntax: log(RT) $\sim $ accuracy * score * difficulty + (1 | subject)

There were also a significant accuracy by difficulty interaction (*β* = 0.097, *t* = 14.34, *p* < 0.001) and accuracy by rest score interaction (*β* = − 0.064, *t* = − 8.76, *p* < 0.001). Correct responses were even slower for more difficult items, but faster for better-performing participants. There was also a significant difficulty by score interaction (*β* = 0.032, *t* = 6.55, *p* < 0.001), such that better-performing participants exhibited even slower responses for more difficult items. This pattern of response time results is visible in Fig. 2c. The three-way interaction term was not significant.

In sum, we found evidence of a speed–accuracy trade-off in performance in the calibration sample. Both correct responses and better-performing participants were slower overall. Notably, better-performing participants demonstrated adjustments to their response times as function of item difficulty; whereas the lowest scoring participants maintained an equivalent work rate regardless of item difficulty, the highest scoring participants showed the largest slowing in responding as items became more challenging. We return to these results in the General discussion.

#### Item response models

The results of the model comparison is summarized in Table 2. Of the first-wave models, which varied only in their specification of item difficulty, Model 3 exhibited the best fit to the data. Indeed, Model 3 demonstrated a considerable improvement in fit over Model 1 (Δ LOCO = 1155.8, se = 60.1) and Model 2 (Δ LOCO = 376.5, se = 34.5). This result indicates the presence of non-negligible residual variance in item difficulty across both item templates and clones. As the best-fitting model in the first wave, Model 3 served as the starting point in the second wave of model fitting.

**Table 2:** Comparison of item response models fit to the MaRs-IB response data

	Difficulty			Discrimination			LOO-CV	
Model	FE-1/2	RE-1	RE-2	FE-1/2	RE-1	RE-2	psis-loco	Δ psis-loco (se)
1	X						27741.4	1326.87 (62.88)
2	X	X					26962.1	547.56 (37.79)
3	X	X	X				26585.5	171.05 (12.65)
4	X	X	X	X			26419.6	5.12 (4.07)
5	X	X	X	X	X		26414.5	–
6	X	X	X	X	X	X	26417.8	3.29 (1.84)

The columns under difficulty and discrimination indicate the specification of those parameters for each model (i.e., the presence of level 1/2 fixed effects, level 1 random effects, and level 2 random effects). LOO-CV values are presented in deviance scale (i.e., smaller values indicate better fit). Abbreviations: PSIS = Pareto-smoothed importance sampling; LOCO = leave-one-cluster-out

Of the second-wave models, which varied only in their specification of item discrimination, Model 5 demonstrated the best fit to the data. In contrast, Model 5 yielded smaller improvements in fit over Model 4 (Δ LOCO = 5.12, se = 4.07) and Model 6 (Δ LOCO = 3.29, se = 1.84). This result suggests the presence of non-negligible residual variance in item discrimination across item templates but not clones. It must be noted these differences in LOCO values from each model to the next were each within two standard errors of the mean, indicating only weak predictive improvement of Model 5 over the others (Vehtari, 2022). As such, we will select Model 5 as the best-fitting model overall, but proceed in discussing it with caution. We also note that all second-wave models exhibited better fits to the data than the first-wave models, supporting the estimation of item discrimination parameters.

#### Aim 1: Quantify the psychometric properties of items in the MaRs-IB

Across all items, the average item difficulty was *μ*_*β*_ = 0.177 (95% HDI = 0.118–0.238). There was considerable variability in item difficulty across items (sd = 1.431, 95% HDI = 1.370–1.492), with the smallest and largest item difficulty parameters spanning a wide range ($\beta _{\min \limits }$ = -3.704, 95% HDI = -4.687 – -2.775; $\beta _{\max \limits }$ = 3.497, 95% HDI = 2.481–4.635). For an average ability participant (*𝜃*_*i*_ = 0), these parameter estimates translate to an expected average accuracy of 58.4% across all items, and ceiling (chance level) performance for the least (most) difficult item.

In turn, the average item discrimination was *μ*_*α*_ = 1.298 (95% HDI = 1.212–1.385). Variability in item discrimination was modest by comparison (sd = 0.221, 95% HDI = 0.121–0.322). Item difficulty and discrimination were uncorrelated across items (*ρ* = -0.060, 95% HDI = -0.379– 0.250). Following convention (Baker and Kim, 2017), the items in the MaRs-IB exhibited medium (*α* ∈ 0.65–1.34) to high (*α* ∈ 1.35–1.69) levels of discrimination and are thus suitable for measuring matrix reasoning ability.

#### Aim 2: Measuring the association between item complexity and functioning

Next, we inspected the associations between item complexty (i.e., number of elements and number of rules) and item functioning. As predicted, a one-unit change in element number was associated with an increase in item difficulty ($\delta _{\beta _{1}}$ = 0.579, 95% HDI = 0.389–0.770), or an approximate 10.6% reduction in accuracy for a participant of average ability. Similarly, a one-unit change in rule number was also associated with an increase in item difficulty ($\delta _{\beta _{2}}$ = 0.514, 95% HDI = 0.378–0.663), or a 9.5% reduction in accuracy. The difference in coefficients was not credibly different than zero ($\delta _{\beta _{1}} - \delta _{\beta _{2}}$ = 0.065, 95% HDI = -0.189 to 0.319), indicating that both attributes exert approximately equal impact on item difficulty.

Together, element number and rule number explained approximately 67.6% of the variance in difficulty across the 64 templates. That is, the majority of variability in difficulty across templates can be attributed to their complexity. Individually the number of elements and number of rules respectively explain 29.5% and 40.6% of the variance in item template difficulty. Across all 384 clones, these two attributes explain 38.6% of the variance in difficulty.

Only the number of rules was associated with item discrimination: a one-unit change in rule number was associated with a marginal increase in discrimination ($\delta _{\alpha _{2}}$ = 0.020, 95% HDI = 0.003–0.037). There was not a credible association between the number of elements and discrimination ($\delta _{\alpha _{1}}$ = -0.015, 95% HDI = -0.036 to 0.008). These two attributes explained 31.9% of the variance in discrimination across templates. Critically, these results must evaluated in light of our parameter recovery analyses (see *Goodness-of-fit* below).

#### Aim 3: Investigating the exchangeability of item clones

Contrary to the assumption that item clones are exchangeable, we found an association between distractor type and item difficulty ($\delta _{\beta _{3}}$ = 1.105, 95% HDI = 0.940–1.269). Items with MD distractors were associated with an estimated 19.9% reduction in accuracy compared to their equivalent items with PD distractors. Together element number, rule number, and distractor type explained slightly over half (52.0%) of the variance in difficulty across item clones. Regardless, the results of the model comparison demonstrated that an item response model which included a clone-level residual variance term for item difficulty was preferred to one without. Together, these two results clearly indicate that not all item clones are equally difficult and, therefore, item clones cannot be assumed to be exchangeable.

**Fig. 3:**
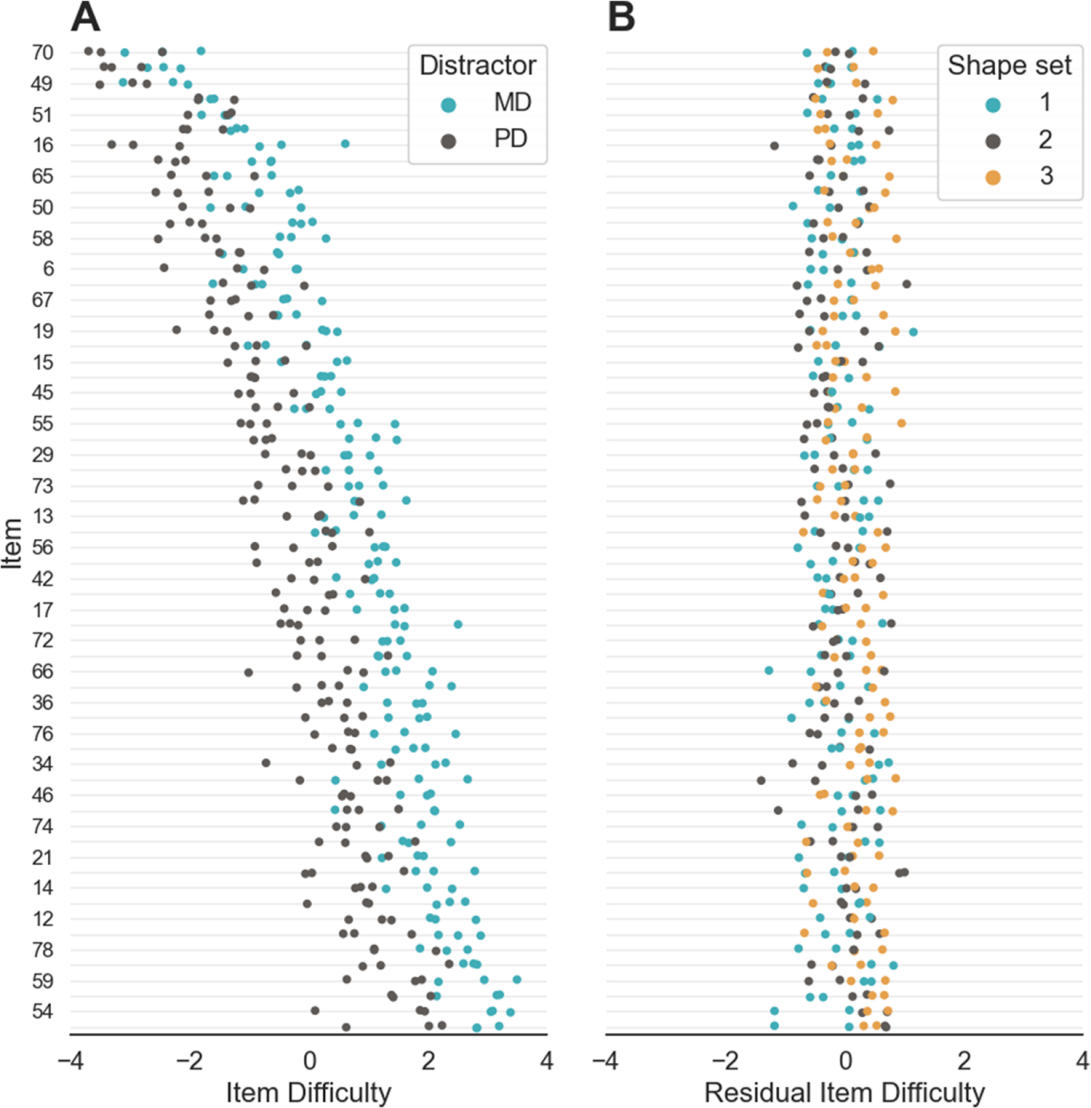
The estimated item difficulty parameters for the items in the MaRs-IB. Each dot represents one item clone. (A) Item difficulty color-coded by minimal difference (MD)-type (*blue*) and paired difference (PD)-type (*grey*) distractors. (B) Residual item difficulty across item clones, after accounting for all other modeled sources of variance, color-coded by shape set

To make clear the residual variance in item difficulty across clones, the estimated item difficulty per clone is presented in Fig. 3. The relative increase in difficulty for items with MD-type distractors compared to PD-type distractors is easily seen (Fig. 3a). In contrast the pattern of residual difficulty across clones, after accounting for all modeled sources of variance, is more complicated (Fig. 3b). The magnitude of the clone-level residual variance is *𝜖*_*βk*_ = 0.620 (95% HDI = 0.538–0.701), or 35.1% of the total variance in difficulty across item clones. This corresponds to a mean absolute difference in difficulty of 0.705 (95% HDI = 0.612–0.786) across clones per item and distractor type, or roughly two-thirds as large as the effect of distractor type. Thus, the residual variability in item difficulty across clones is large, complex, and likely reflects the idiosyncratic contributions of perceptual features.

Turning to item discrimination, we did not find a credible association between discrimination and distractor type ($\delta _{\alpha _{3}}$ = -0.029, 95% HDI = -0.065–0.005). Moreover, a model that included a clone-level random effects term for item discrimination was not preferred to a model without. As such, there is not sufficient evidence to reject the assumption that item clones are equivalently discriminating. We note this result should be interpreted with caution given the relatively weak predictive improvement of the best-fitting model compared to a model with a clone-level random effects term.

Finally, we inspected the relationship between mean response time and item functioning. As expected, there was a positive association between mean response time and item difficulty ($\delta _{\beta _{4}}$ = 0.541, 95% HDI = 0.414–0.671) indicating response times were slower for more difficult items. There were not a credible association between item discrimination and average response time ($\delta _{\alpha _{4}}$ = -0.010, 95% HDI = -0.030– 0.009).

#### Person-level effects

In the best-fitting model, there were several credible associations between person-level attributes and ability. There was a negative association between age and ability (*ρ*_1_ = -0.299, 95% HDI = -0.353–0.246). There was also a small association between gender and ability (*ρ*_2_ = 0.128, 95% HDI = 0.078–0.182), such that women performed marginally better than men. Average response time was positively associated with ability (*ρ*_3_ = 0.427, 95% HDI = 0.374–0.478), as was Δ response time (*ρ*_4_ = 0.368, 95% HDI = 0.313–0.424). In other words participants with higher levels of ability spent more time on each item on average, and even longer for more difficult items. Together, these four person attributes explained 50.3% of the variance in ability. These results are consistent with a speed–accuracy trade- off in performance. That is, higher levels of ability in this sample reflect, in part, a tendency to slow down and respond more carefully (especially for the most challenging items).

#### Goodness-of-fit

We next evaluated the fit of the best-fitting model to the data using two posterior predictive model checking measures. The PPP value for chi-square discrepancy statistic did not exceed the critical threshold ($\chi ^{2}_{NC}$ = 15.904, PPP value = 0.297), indicating that the model was sufficiently able to reproduce the distribution of observed scores. The PPP value for the SGDDM statistic did exceed the critical threshold (PPP value = 0.019). However, the mean of the realized SGDDM values (SGDDM_*r*_ = 0.0180) was only marginally larger than the mean of the posterior predicted SGDDM values (SGDDM_*p*_ = 0.0175). This means that the residual inter-item correlations in the data were small on average and only slightly larger than what we would expect to observe by chance. As such, there is little evidence for local dependence in the data. Overall then, we can conclude the best-fitting model provides an adequate fit to the data.

Finally, we inspected the results of the parameter recovery analysis (the complete results are reported in the Supplementary Materials). Briefly, we found we were able to recover item difficulty parameters with excellent precision. Conversely, we observed only adequate recovery of the item discrimination parameters. The results may in turn explain why we detected only one credible association between item attributes and item discrimination. Although we were adequately-powered to detect large associations between item attributes and discrimination (i.e., explaining 14% or more variance in the latter), we are poorly powered to detect smaller associations. This was not true for item difficulty, where we were well-powered to detect associations of the magnitude reported above. Importantly, we found through follow-up analyses that the less-than-perfect recovery of item discrimination parameters yielded only negligible effects on the reliability of test forms produced using optimal test assembly procedures. Therefore, our analyses were sufficiently powered for the primary objectives of this study.

### Discussion

In this first study, we recruited a large sample of adults to complete items from the MaRs-IB, making sure that each item template and clone were completed by a sizable number of participants. Using multilevel item structure models, we then estimated the psychometric properties (i.e., difficulty and discrimination) of each item. Corroborating the results of an initial investigation of the MaRs-IB (Chierchia et al., 2019), we found that the items in the MaRs-IB vary greatly in their difficulty—a prerequisite for measuring nonverbal reasoning across the spectrum of ability. We also found that the items in the MaRs-IB exhibited medium-to-large levels of discrimination, similar to what has been found in investigations of other matrix reasoning tasks (Chiesi, Ciancaleoni, Galli, & Primi, 2012; Chiesi, Ciancaleoni, Galli, Morsanyi, & Primi, 2012; Elst et al., 2013).

We also investigated how item complexity shapes item functioning. We found that element number and rule number were both positively associated with item difficulty, the effects of which were of approximately equal magnitude (i.e., a one-unit change in either was independently associated with a roughly 10% reduction in performance). This finding is interesting in light of previous investigations of nonverbal reasoning tasks, which have found a greater influence on item difficulty from either the number of elements (Bethell-Fox, Lohman, & Snow, 1984) or number of rules (Mulholland, Pellegrino, & Glaser, 1980). One possible reason for this discrepancy is that these two attributes are largely uncorrelated across items in the MaRs-IB, which allows for unconfounded estimates of their effects. Together element number and rule number explained 67.6% of the variance in difficulty across templates (and 38.6% of the variance across item clones), which is in line with previous investigations of matrix reasoning tasks (Carpenter, Just, & Shell, 1990; Matzen, Van Der Molen, & Dudink, 1994). These findings further validate the design of the MaRs-IB insofar that item complexity is a primary determinant of item difficulty. Finally, we found that rule number, but not element number, was associated with item discrimination. However, this finding must be interpreted with caution as our parameter recovery analysis revealed that our sample size was adequately powered to detect only larger effects (attributes explaining 14% or more variance in item discrimination). Future studies with larger samples will be required to more thoroughly investigate the relationship between item complexity and discrimination.

Finally, we investigated whether the item clones in the MaRs-IB are psychometrically equivalent and exchangeable. Crucially, we found evidence that item clones are not equally difficult. Unexpectedly, and contrary to the results of Chierchia et al., (2019), distractor type emerged as a robust predictor of item difficulty (i.e., items with MD-type distractors are associated with a 19.9% reduction in accuracy compared to the same items with PD-type distractors). This discrepancy in findings between studies may reflect the much larger number of responses available per clone in our study. We also found non-negligible residual variability in item difficulty across clones that was difficult to characterize and likely reflects the effects of low-level perceptual features. Together, our results clearly indicate that item clones in the MaRs-IB cannot be assumed to be psychometrically equivalent and should not be treated as exchangeable.

In summary, the results of the calibration study confirm the MaRs-IB to be a useful and psychometrically valid resource for measuring matrix reasoning ability. The items vary greatly in their difficulty, and possess medium-to-large levels of discrimination, thereby making the MaRs-IB suitable for measuring matrix reasoning across a wide range of ability. That item clones are not equivalently difficult, and therefore should not be treated as exchangeable, does not invalidate this conclusion. The item parameter estimates derived as part of this study are therefore suitable for use in designing new MaRs-IB measures with both good reliability and potential for reuse, as we demonstrate in the next study.

## Validation study

### Objectives

The purpose of the second study was to provide a demonstration of how to use the item parameter estimates, derived in the previous study, to design new MaRs-IB measures. Specifically, we use optimal test assembly (Van der Linden, 1998) to design test forms that maximize test reliability given researcher-defined constraints.

### Optimal test assembly

Given the starting point of 64 item templates with six clones each, there is a vast number of possible MaRs-IB test forms we could construct. Returning to our motivating problem—the availability of matrix reasoning measures with the potential for reuse—we decided to construct two new sets of test forms: a set of three short-form measures and a set of two long-form measures.

To design the test measures, we used mixed integer programming (Van der Linden, 2005) in order to maximize the test information function (TIF) of each test subject to the following constraints:

1. Each short- and long-form test was required to contain 12 and 24 items, respectively. This was chosen to minimize the administration time of a given form while achieving a minimal score reliability ≥ 0.7 and ≥ 0.8.
2. Within each test set, the difference in TIFs between tests should be minimized. This constraint was adopted to ensure that each test form had approximately equal psychometric properties (i.e., equivalent reliability across ability levels).
3. For a given test, clones selected for inclusion could either use MD-type or PD-type distractors, but not both. This constraint was adopted in order to prevent item redundancy (i.e., including the same puzzle twice in one test form).
4. All three short-form tests (but not the long-form tests) were required to be made up of clones from the same items. This constraint was adopted in order to maximize the similarity of the short forms.

For both assembly procedures, the TIF was maximized at five ability levels (*𝜃* = -1.0, -0.5, 0.0, 0.5, 1.0), which previous simulation studies have shown to be generally sufficient (Van der Linden, 2005). Solutions to the mixed integer programming problem were found using the *mip* python package (v1.13.0; Santos and Toffolo, 2020).

**Fig. 4:**
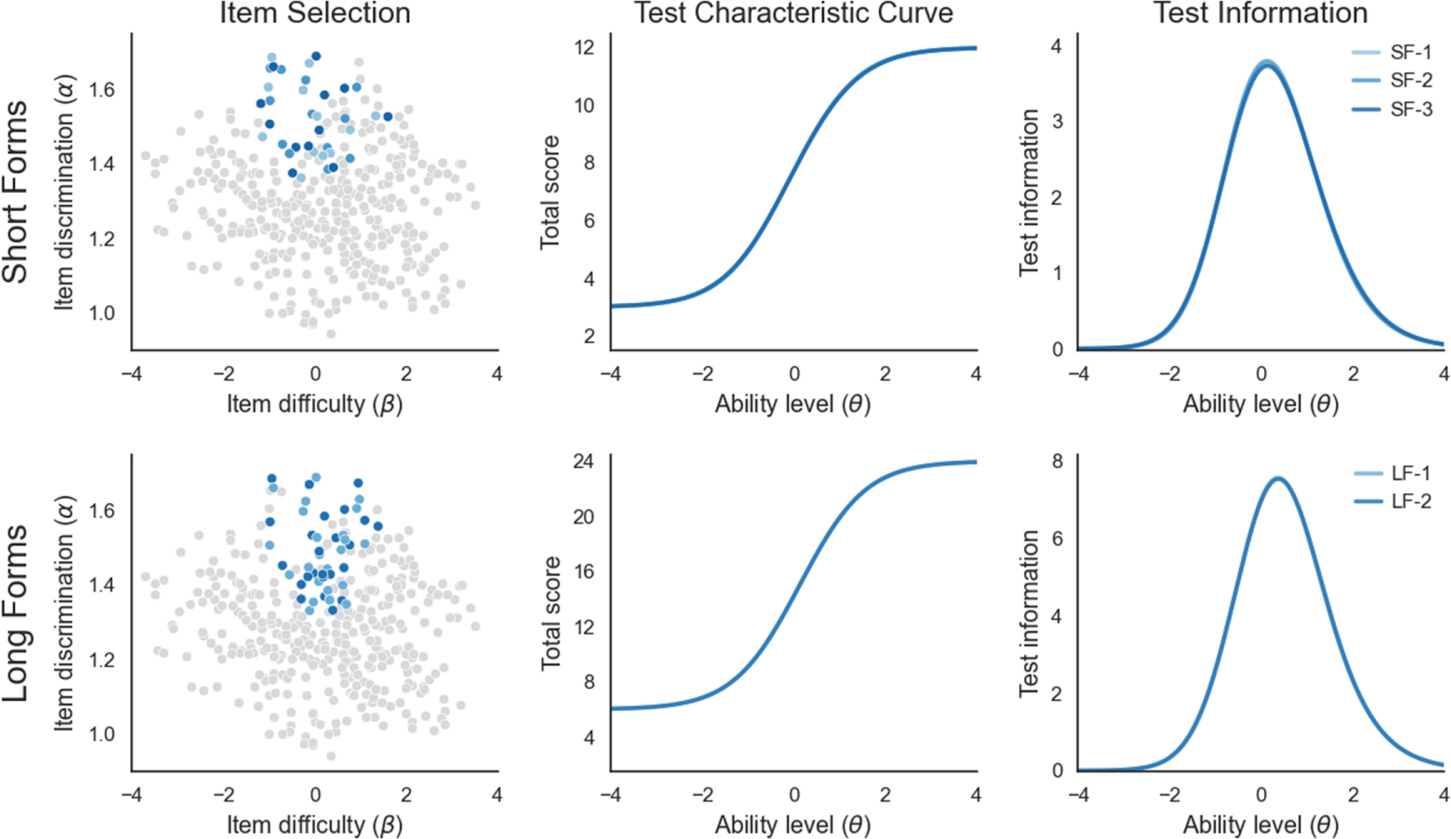
Results from the test assembly of three parallel MaRs-IB short form (SF) measures. (A) The psychometric properties of the items selected for each form, compared to all remaining items. Points represent the posterior means of the item difficulty and discrimination parameters. (B) The test characteristic curves (TCCs) for each short form. The degree of overlap highlights the similarity of expected scores for each test form. (C) The test information functions (TIFs) for each short form. The degree of overlap highlights the similarity of reliability for each test form

The results of the test assembly are presented in Fig. 4. As expected, the MIP solver selected for items that were more discriminating on average (Fig. 4, left column). Though this was not an explicit constraint, the test characteristic curves (TCCs) for each test form, within a set, were markedly similar (Fig. 4, middle column). Based on the TCCs, the expected average score was 7.7 out of 12 items for the short-form measures and 14.4 out of 24 items for the long-form measures. (Note that, because the lowest ability participants are expected to guess an item correctly 25% of time, the lower asymptote of the TCCs is one quarter of the maximum possible score.) Finally, the TIFs for each test form within a set are nearly identical (Fig. 4, right column), as would be expected given the last assembly constraint. We should therefore expect each test form within a set to have approximately equal reliability in measuring matrix reasoning ability. Indeed, the IRT test score reliability coefficients (Kim & Feldt, 2010; Nicewander, 2018) for each short form were similar (short form 1: *ρ* = 0.706; short form 2: *ρ* = 0.709; short form 3: *ρ* = 0.705), as were the coefficients for each long form (long form 1: *ρ* = 0.821; long form 2: *ρ* = 0.821).

Next, we proceeded to administer each test form to a second sample of participants. Our aim was to verify that observed scores on each test form conformed to our expectations based on the TCCs and TIFs. Doing so would provide evidence that we were successful in calibrating the functioning of the items in the MaRs-IB in the previous study. We also sought to demonstrate convergent validity by correlating participants’ total scores on the MaRs-IB test forms with their total scores on an abbreviated version of the Raven’s progressive matrices (Bilker et al., 2012).

### Methods

#### Participants

Two samples of participants (*N*_1_ = 347, *N*_2_ = 360) were recruited from the Prolific Academic platform to participate in an online behavioral experiment in November, 2021 and September, 2022. Participants were eligible if they currently resided in the United States and had not participated in the calibration study. Study duration was on average 10.8 minutes (sd = 4.6) for sample 1 and 14.3 minutes (sd = 4.9) for sample 2. Participants received monetary compensation for their time (rate USD $10/hr) plus a bonus up to $0.75 (sample 1) or $1.50 (sample 2) based on task performance. Sample 1 earned on average $2.10 USD (sd = $0.18) and sample 2 earned on average $3.17 USD (sd = $0.30). This study was approved by the Institutional Review Board of Princeton University (#7392), and all participants provided informed consent.

To ensure data quality, the data from multiple participants were excluded prior to analysis (see Exclusion Criteria below) leaving the data from a total of 600 participants (*N*_1_ = 300, *N*_2_ = 300) for analysis. Across samples, the majority of participants identified as women (men: *N* = 281; women: *N* = 281; non-binary or other: *N* = 25; rather not say: *N* = 2). Participants were on average 36.6 years old (sd = 13.1; range, 18–76). The combined sample was relatively well educated, with the majority having completed a bachelor’s degree (*N* = 227) or master’s degree or higher (*N* = 76). By comparison, fewer participants endorsed having completed only some college (*N* = 142), only a high school degree or lower (*N* = 98), or preferred not to say (*N* = 1).

#### Procedure

The study was divided into three parts. After providing consent, participants first completed three short surveys: the 10-item need for cognition survey (Chiesi, Morsanyi, Donati, & Primi, 2018), the 8-item PROMIS Cognitive Function-Short Form (8a) (Iverson, Marsh, Connors, & Terry, 2021), and the 8-item subject numeracy scale (Fagerlin et al., 2007). These measures were included as part of exploratory analyses to measure the associations between personality and matrix reasoning ability (these correlations are reported in Table S2 of the supplementary materials). Afterwards participants completed either one of the three 12-item MaRs-IB short forms (sample 1) or one of the two 24-item MaRs-IB long forms (sample 2). The administration procedure of the test forms was identical to that in the previous study. Finally, all participants completed the 9-item abbreviated Raven’s progressive matrices (RPM form A; Bilker et al., 2012). To maximize consistency in administration, the presentation format and instructions for the RPM task were the same as for the MaRs-IB test forms.

#### Exclusion criteria

To ensure data quality, the data from multiple participants were excluded prior to analysis for one or more of the following reasons: failing to complete all three sections of the experiment (*N* = 27); failing one or more attention checks (Zorowitz, Niv, & Bennett, 2021) embedded in the self-report measures (*N* = 49); experiencing technical difficulties during the experiment (*N* = 24); rapid guessing on four or more items (*N* = 24); or failing to respond on four or more items (*N* = 2). In total, 107 of 707 (15.1%) participants were excluded leaving the data from *N* = 600 participants for analysis.

#### Analysis

We calculated descriptive statistics to summarize the distribution of participants’ total scores on the MaRs-IB short forms, MaRs-IB long forms, and abbreviated RPM. The convergent validity between the MaRs-IB and RPM measures was estimated by calculating the Pearson correlation between participants’ total scores on those measures (collapsing across MaRs-IB test form). These calculations were performed separately for the MaRs-IB short-form and long-form measures.

To quantify the agreement between the observed and expected MaRs-IB scores, we relied on posterior predictive model checking. Specifically, we calculated a $\chi ^{2}_{NC}$ discrepancy measure (Sinharay et al., 2006), which compares the observed and model-predicted proportion of participants at each total score level. The model-predicted scores were obtained via a two-step procedure. First, we estimated for all participants a posterior distribution of latent ability parameters under the 3PL model, given their observed item responses and the item parameters estimated during the calibration study. Second, using the distribution of ability estimates, we generated a sample of predicted responses. We computed a posterior predictive *p* (PPP) value based on the discrepancy statistic, where a poor fit to the data is indicated when the PPP value is extreme (PPP ≤ 0.05). This procedure was also repeated for the abbreviated RPM measure, but the item difficulty and discrimination parameters were jointly estimated alongside the person ability parameters under the 3PL model. The item guessing parameters were held fixed to their nominal guessing rates.

The latent ability and RPM item parameters were estimated within a Bayesian framework using Hamiltonian Monte Carlo as implemented in Stan (v2.22) (Carpenter et al., 2017). Four separate chains with randomized start values each took 7500 samples from the posterior. The first 5,000 samples from each chain were discarded. As such, 10,000 post-warmup samples from the joint posterior were retained. The $\hat {R}$ values for all parameters were less than 1.01, indicating acceptable convergence between chains, and there were no divergent transitions in any chain. The prior for the latent ability parameters was Normal(0,1). For the RPM response model, the prior on the item difficulty and discrimination parameters were Normal(0.0, 2.5) and Lognormal(1.0, 1.0), respectively.

### Results

Performance on the MaRs-IB test forms is summarized in Table 3. On average, participants completed the short-form measures in 174 s (sd = 54.0 s) out of a possible 360 s total and correctly solved 8.0 of 12 items (sd = 2.5). In turn, participants completed the long-form measures in 349 s (sd = 97.9 s) out of a possible 720 s total and correctly solved 15.7 of 24 items (sd = 5.1). A one-way ANOVA comparing the total scores across the three short forms was not statistically significant (F(2,297) = 0.253, p = 0.777); so too, the distributions of total across the two long forms were not significantly different (F(1,298) = 0.219, p = 0.640). Crucially, none of the posterior predictive *p* values corresponding to the $\chi ^{2}_{NC}$ discrepancy measure exceeded the critical value, indicating that the previously estimated item parameters were sufficiently able to predict the observed distribution of total scores for each test form. In other words, performance on each new MaRs-IB test form conformed to expectations, indicating that item functioning was well calibrated in the previous study (Table 2).

**Table 3:** Summary of performance on the MaRs-IB 12-item short forms (SF), MaRs-IB 24-item long forms (LF), and Raven’s progressive matrices (RPM) 9-item short form (SF) measures

Measure	#	Sample	Task time (sd)	Mean score (sd)	IQR	$\chi ^{2}_{NC}$ (ppp)	Reliability
MaRs-IB SF	1	*N* = 103	174.9 s (53.3)	7.9 (2.4)	7 - 10	13.6 (0.284)	0.706
	2	*N* = 98	169.0 s (53.0)	8.2 (2.6)	7 - 10	6.6 (0.811)	0.709
	3	*N* = 99	178.5 s (55.7)	7.9 (2.7)	6 - 10	7.1 (0.778)	0.705
MaRs-IB LF	1	*N* = 153	351.7 s (92.6)	15.9 (4.9)	13 - 20	12.5 (0.910)	0.821
	2	*N* = 147	346.8 s (103.4)	15.6 (5.4)	11 - 20	29.7 (0.120)	0.821
RPM SF	A	*N* = 600	126.7 s (39.6)	4.5 (2.0)	3 - 6	10.3 (0.359)	0.613

Reliability reflects the IRT test score reliability coefficient. Abbreviations: IQR = interquartile range

**Fig. 5:**
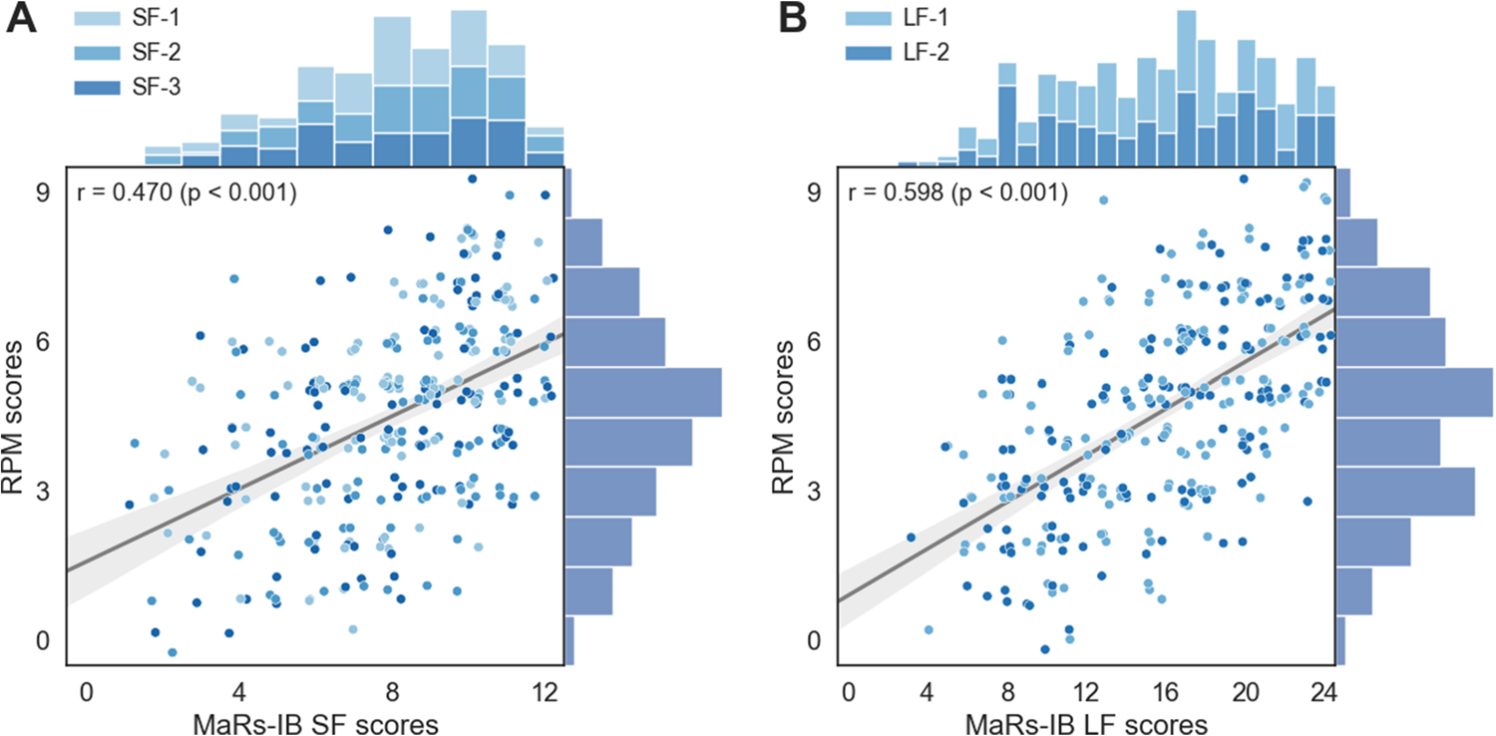
The joint distribution of total scores on the abbreviated Raven’s progressive matrices (RPM) and the MaRs-IB test forms. (A) Scores from the MaRs-IB 12-item short forms (SF). (B) Scores from the MaRs-IB 24-item long forms (LF). The distribution of observed scores for each measure are plotted in the margins

Performance on the abbreviated RPM measure is also summarized in Table 3. On average, participants completed the measure in 127 s (sd = 39.6 s) and responded correctly on 4.5 of 9 items (sd = 2.0). The proportion of correct responses on the RPM was significantly lower than that observed for the MaRs-IB short forms (*t* = 13.028, *p* < 0.001) and long forms (*t* = 12.812, *p* < 0.001), indicating that the RPM is more difficult. Notably, the calculated IRT test reliability for the abbreviated RPM measure was smaller than that for both the MaRs-IB short- and long-form measures. The average correlation between scores on the RPM and MaRs-IB short-form measures was *r* = 0.470 (*p* < 0.001); between the RPM and long-form measures, the correlation was *r* = 0.598 (*p* < 0.001; Fig. 5). These results indicate satisfactory convergent validity between the two families of matrix reasoning measures.

To summarize, we used the estimated item parameters and optimal test assembly procedures to construct two new sets of MaRs-IB test forms. These new measures were quick to administer, produced score distributions consistent with model predictions, possess adequate-to-good test score reliability, and demonstrated convergent validity with an established measure of matrix reasoning ability. Furthermore, all of these new test forms were more reliable than an abbreviated version of the Raven’s progressive matrices. Together, these results demonstrate our success in calibrating the functioning of MaRs-IB items and also the feasibility of constructing new reliable tests of matrix reasoning with the MaRs-IB.

## General discussion

Here we have provided a comprehensive investigation of the MaRs-IB. Using item structure models fit to data collected from a large sample of adult participants, we found that the MaRs-IB possesses many desirable psychometric properties. The items in the MaRs-IB span a considerable range of difficulty and exhibit medium-to-high levels of discrimination. The MaRs-IB is therefore suitable for measuring matrix reasoning across the ability spectrum in the general adult population. We also verified that the design of the MaRs-IB was, at least in part, successful. Item complexity (i.e., element number and rule number) was positively associated with, and explained the majority of variance of, item difficulty. In contrast, we also uncovered some undesirable properties of the MaRs-IB. Namely, we observed substantial variability in item difficulty (both systematic and unsystematic) across item clones. As a consequence, item clones in the MaRs-IB cannot be assumed to be psychometrically equivalent and should not be treated as exchangeable.

Using the item parameters estimates, we then constructed two new sets of MaRs-IB test forms using optimal test assembly methods. These test measures were designed to be maximally reliable under differing constraints (e.g., administration times of 2–4 min vs. 4–8 min). In a second sample of participants, we found that the total scores from each test form was consistent with model predictions and predictive of performance on a second, established measure of matrix reasoning ability. Collectively, these results highlight the success of the item calibration study in producing accurate estimates of item functioning for the items in the MaRs-IB.

Of additional note are the associations we found between accuracy and response time. We found that correct responses and better-performing participants were slower on average, consistent with a speed–accuracy trade-offs in performance (Heitz, 2014). Interestingly, we also found an interaction between participant ability and item difficulty: whereas the worst-performing participants maintained the same work rate irrespective of item difficulty, the best-performing participants spent more time deliberating as items became more challenging. These results are consistent with a persistence interpretation of ability, wherein good task performance in part reflects a willingness to invest time in the solution process and poor task performance reflects a tendency to give up sooner (Ranger, Kuhn, & Pohl, 2021). As such, the MaRs-IB may be suitable not only for measuring matrix reasoning ability but also mental effort costs (Kool & Botvinick, 2018), opportunity costs (Payne, Bettman, & Luce, 1996), or other motivational factors (Duckworth et al., 2011) related to people’s tendency to exert effort or give up. The use of more sophisticated models (e.g., Ranger & Kuhn, 2014) may help to disentangle the relative contributions of latent ability and persistence to performance on the MaRs-IB.

The current investigation of the MaRs-IB is not without its own limitations. One notable limitation is our sample. Here we analyzed response data collected from an online adult sample which was relatively young and well-educated. We cannot guarantee that the psychometric properties of the MaRs-IB reported here will generalize to other populations or testing contexts. Future researchers should consider replicating the current study in other populations of interest (e.g., children, clinical samples). By using item response models in this study, however, we make possible the opportunity for future IRT “linking” studies. IRT linking describes a set of methods to establish the comparability of item and ability parameters estimated from response data collected from two or more groups that differ in ability (Lee & Lee, 2018). Future studies might exploit these methods to provide new insights, not only in how the functioning of the MaRs-IB may differ in across populations, but also in how matrix reasoning ability changes across populations. Future studies involving larger samples could also provide populations norms for MaRs-IB test scores, which would enable researchers to compare the outcomes of specific individuals or groups (e.g., clinical groups) against the performance of the general population (of a particular country or region).

A second limitation of the present analyses is our relatively narrow investigation of the sources of variance in item functioning in the MaRs-IB. Previous psychometric investigations of the Raven’s progressive matrices, for example, have highlighted the impact of not only the number of rules but also the types of rules on item difficulty (Carpenter et al., 1990; Embretson, 1998). So too, it is possible that the four rules in the MaRs-IB are not equally difficult or discriminating (e.g., color changes may be easier to process than position changes) and may explain part of the residual variance in item functioning across item templates. Other studies of matrix reasoning tasks have identified large contributions of perceptual features to item difficulty (Primi, 2001; 2014), effects which were not explicitly considered here. Future studies should consider investigating these and other sources of variance in item functioning—a possibility made easier by the public release of all the data collected as part of the current studies.

In conclusion we have provided the most comprehensive psychometric validation of the MaRs-IB, the current largest available bank of open access matrix reasoning items, finding that it is suitable for measuring matrix reasoning ability in the general adult population. We hope that the results and materials presented here will encourage researchers to design their own matrix reasoning tests, tailored to their needs, using the MaRs-IB. In support of this, we have made all of our data, code, and model outputs available at: https://github.com/ndawlab/mars-irt.

### Supplementary Information

ESM 1 (PDF 559 KB)

## Footnotes

1 We did not collect new data for the 16 easiest items in the MaRs-IB (items 1, 2, 3, 4, 5, 7, 8, 9, 32, 33, 38, 41, 43, 48, 57, 68) because performance on these items was at or near ceiling.

2 A threshold of 3 s was decided on based on pilot data. See the Supplementary Materials for details.

3 Removing mean response time as a clone-level attribute did not appreciably change the estimated item parameters; see Fig. S1.

4 These models still included a fixed guessing parameter (*γ* = 0.25). We also estimated models with no guessing parameter (i.e., one-parameter logistic or Rasch models), but model comparison indicated these models provided worse fits to the data (Table S1).
