# Supplementary material for: An item response theory analysis of the matrix reasoning item bank (MaRs-IB)
Source: Behav Res Methods. 2023 Apr 5;56(3):1104–22. doi: 10.3758/s13428-023-02067-8 (PMC10551052; doi:10.3758/s13428-023-02067-8)
Supplement: Supplementary file 1 — (PDF 559 KB) [file 13428_2023_2067_MOESM1_ESM.pdf]

Supplemental materials for "An item response theory analysis of the Matrix Reasoning Item Bank (MaRs-IB)"

Samuel Zorowitz[1], Gabriele Chierchia[3], Sarah-Jayne Blakemore[3], Nathaniel D. Daw[1,2]

[1]Princeton Neuroscience Institute, Princeton University, USA

[2]Department of Psychology, Princeton University, USA

[3]Department of Psychology, University of Cambridge, Downing Street, Cambridge, UK

Supplemental materials for "An item response theory analysis of the Matrix Reasoning Item Bank (MaRs-IB)"

Speed-accuracy trade-offs in Chierchia et al. (2019)

To investigate the possibility of speed-accuracy trade-offs in the MaRs-IB response data collected by Chierchia et al. (2019), we looked at the proportion of correct responses to the easiest items (dimension 1 & 2 items) as a function of the number of participants having reached that item. The logic is that, if items that appeared later in the fixed-order test were disproportionately reached by participants sacrificing accuracy for speed, then we should observe a positive correlation between the total number of available responses and proportion correct amongst the easiest items.

We detected strong positive correlations between proportion correct and number reached (dimension 1 items: $\rho = 0.514$, p = 0.050; dimension 2 items: $\rho = 0.767$, $p < 0.001$; combined: $\rho = 0.695$, $p < 0.001$). This result supports the hypothesis that participants that did reach items later in the test did so by prioritizing speed at the expense of accuracy. This suggests that the summary statistics of later items released as part of Chierchia et al. (2019) are likely biased indicators of item difficulty.

Defining a threshold for rapid guessing

In online testing environments, it is inevitable that some participants will not engage meaningfully with an experiment and instead in engage in careless or insufficient effort responding. On matrix reasoning tasks, one such low-effort strategy is rapid guessing wherein participants response in such a short time that there is no way they could have meaningfully considered an item (Wise, 2017). In sufficient quantities, the presence of rapid guesses in data can systematically bias estimates of item parameters. Thus, if possible, rapid guess responses should ideally be identified and removed.

There are a number of approaches for identifying rapid guess responses (for a review, see Wise (2017)). We opted for a threshold approach, in which responses taking less than a particular time would be denoted as rapid guesses and participants exhibiting too many rapid guessing responses would be excluded from the data. To

define this threshold, we fit an extended version of the effort-moderated item response theory (EM-IRT) model (Wise & DeMars, 2006) to a small dataset of responses collected during piloting. In the EM-IRT, the probability of correct responding for participant i to item j is defined as the following mixture:

$$p(y_{i,j} = 1) = (1 - w_{ij}) \cdot \gamma + w_{ij} \cdot \text{logit}^{-1} (\alpha_j \cdot \theta_i - \beta_j)$$

where θ_i is the latent ability for person i, and β_j, α_j, and γ_j are the difficulty, discrimination, and guessing parameters for item j. As in the main text, here we fixed the guessing parameter for every item clone to the nominal guessing rate ($\gamma_j = 0.25$). Crucially, w_{ij} is a weight parameter, bounded between zero and one, that controls whether a participant is responding effortfully in accordance with their ability ($w_{ij} \to 0$) or engaging in rapid guessing responding ($w_{ij} \to 1$).

Here we defined the rapid guessing weight as a function of participant's response time on that trial:

$$w_{ij} = \text{logit}^{-1} \left(\zeta_0 + z_{ij} \cdot \zeta_1 + z_{ij}^2 \cdot \zeta_2 \right)$$

where z_{ij} is the (log-transformed) response time for participant i for item j, and ζ_n are regressing coefficients mapping responses times to rapid guessing weights. Thus, this form of the EM-IRT model learns a function in a data-driven fashion to classify responses as having originated from effortful or rapid guessing response strategies.

We fit this model to data collected from a total of N=180 participants recruited from the Prolific Academic platform as part of a pilot experiment (independent of the experiments presented in the main text). Each participant completed one of two sets of eight items from the MaRs-IB. The EM-IRT model estimated within a Bayesian framework using Hamiltonian Monte Carlo as implemented in Stan (v2.22) (Carpenter et al., 2017). Four separate chains with randomised start values each took 3,000 samples from the posterior. The first 2,000 samples from each chain were discarded. As such, 4,000 post-warmup samples from the joint posterior were retained. The $\hat{R}$ values for all parameters were equal to or less than 1.01, indicating acceptable convergence

between chains, and there were no divergent transitions in any chain.

The estimated weights (w_{ij}) across all subjects and items are plotted as a function of their corresponding response time in Figure S3. As can be observed, the weights quickly approach 1 for response times faster than 5 seconds. Interestingly, weights begin to rise again for responses after 20 seconds suggesting that participants have an internal awareness of the amount of time that has elapsed.

To define a threshold for rapid guessing, we found the corresponding response time for which $w = 0.5$. This was at approximately 3 seconds. Thus, in the main experiments we defined rapid guessing as responses taking fewer than 3 seconds.

Organization of geometric stimuli in the MaRs-IB

Every item template in the MaRs-IB has three unique versions that differ only in the geometric shapes populating its cells. There are 45 unique geometric shapes in total, organized into nine stimulus sets, where each item clone draws a subset of shapes from one of the nine sets (Figure S2). As such, shape set — indicating whether a clone is the 1st, 2nd, or 3rd version of an item template — is not a meaningful nominal variable. Furthermore, item clones drawing from the same shape set are not always populated by the same geometric shapes, making complicated the possibility of modeling the nine stimulus sets instead. The most rigorous way of modeling the presence or absence of a given geometric shape on item functioning would be to include each as a binary attribute predicting item difficulty and discrimination. In order to keep our models simple, we elected to leave shape set unmodeled and accounted for by the residual variability terms instead.

Bayesian additive multilevel item structure (AMIS) model priors

In the additive multilevel item structure (AMIS) models, there are three families of parameters: item difficulty parameters, item discrimination parameters, and person ability parameters. We describe the priors for each set of parameters in turn. The model code is publicly available at https://github.com/ndawlab/mars-irt.

The item difficulty parameters can be decomposed into their fixed and random effects components. The fixed effects components include the grand mean, μ_β, the effects of template-level (level-1) attributes, δ_{β_n}, and the effects of clone-level (level-2) attributes, δ_{β_m}. All three fixed effects terms were assigned standard normal priors, $\mathcal{N}(0, 1)$. The template- and clone-level random effects components, $\epsilon_{\beta j}$ and $\epsilon_{\beta k}$, were each assigned normally distributed priors with a mean of zero and standard deviations estimated by the model, $\sigma_{\beta j}$ and $\sigma_{\beta k}$. These standard deviations were, in turn, assigned half-student-t distributions with a mean of zero, a standard deviation of one, and three degrees of freedom, $\text{StudentT}(3, 0, 1)$.

Similarly, the item discrimination parameters can be decomposed into their fixed and random effects components. The fixed effects components include the grand mean, μ_α, the effects of template-level (level-1) attributes, δ_{α_n}, and the effects of clone-level (level-2) attributes, δ_{α_m}. All three fixed effects terms were assigned standard normal priors, $\mathcal{N}(0, 1)$. The template- and clone-level random effects components, $\epsilon_{\alpha j}$ and $\epsilon_{\alpha k}$, were each assigned normally distributed priors with a mean of zero and standard deviations estimated by the model, $\sigma_{\alpha j}$ and $\sigma_{\alpha k}$. These standard deviations were, in turn, assigned half-student-t distributions with a mean of zero, a standard deviation of one, and three degrees of freedom, $\text{StudentT}(3, 0, 1)$. Importantly, item discrimination parameters were restricted to be in the range $\alpha_{jk} \in [0, 5]$. This was achieved by passing the clone-level discrimination parameter through the standard normal cumulative distribution function (i.e. scaling parameters between 0 and 1), and multiplying the result by 5.

Finally, the person ability parameters can be decomposed into their fixed and random effects components. The fixed effects components comprise the partial correlation coefficients, ρ_p. Before transformation, the partial correlation coefficients were assigned standard normal priors, $\mathcal{N}(0, 1)$. These values were then transformed such that the sum of their squared values could not exceed 1. The random effects components, ϵ_θ, were assigned a normally distributed prior with a mean of zero and a variance equal to 1 - minus the sum of squared partial correlations (i.e. fixing the

variance of the person ability distribution to 1).

Parameter recovery & power analysis

In order to assess the robustness of our item calibration analysis, we performed a parameter recovery and power analysis. The objectives of this analysis were threefold. First, we wanted to evaluate our ability to accurately estimate person and item parameters given our sample size and experiment design. Second, we wanted to quantify our power to detect an association between item attributes and item functioning (i.e. difficulty, discrimination) for different magnitudes of association. Third, we wanted to determine how imprecision in our estimates of item parameters impact optimal test assembly and test score reliability.

We generated 100 artificial datasets whose statistical properties were matched to what we observed empirically in the item calibration dataset. Specifically, each dataset was composed of simulated item responses from 1500 "participants" whose latent abilities were drawn from a standard normal distribution. Each participant "completed" 16 item clones from a pool of 384 item clones (nested among 64 item templates). Thus, each artificial item clone was completed by an average of 62.5 participants. The simulated distribution of item difficulty and discrimination parameters were matched to what was observed empirically ($\mu_\beta = 0.20$, $\sigma_\beta = 1.55$; $\mu_\alpha = 1.30$, $\sigma_\alpha = 0.22$). The total variance explained by item attributes for item difficulty and discrimination parameters was also matched to what was observed empirically ($r^2_\beta \approx 60\%$; $r^2_\alpha \approx 40\%$). To estimate our statistical power to detect associations between item attributes and parameters, we specified two large effects and two small effects per item parameter. That is, there were two pairs of attributes that explained 20% and 10% of the variance in item difficulty, respectively; and there were two pairs of attributes that explained 14% and 7% of the variance in item discrimination, respectively. Based on what we observed empirically, the residual variance in item difficulty and discrimination parameters was split equally between the template- (ϵ_j) and clone-levels (ϵ_k).

Models 5 and 6 were fitted to each simulated dataset. We tested Model 5 because

this was the model deemed the best-fitting, parsimonious model in the item calibration study. We tested Model 6 because it is true data generating model for the simulations. Comparing these two models therefore allows us to evaluate whether selecting Model 5 produces any systematic bias in our analyses. All models were estimated within a Bayesian framework using Hamiltonian Monte Carlo as implemented in Stan (v2.22). For all models, four separate chains with randomised start values each took 4,000 samples from the posterior. The first 3,000 samples from each chain were discarded. As such, 4,000 post-warmup samples from the joint posterior were retained. The $\hat{R}$ values for all parameters were less than 1.02, indicating acceptable convergence between chains, and there were no divergent transitions in any chain.

The results of the parameter recovery study are summarized Figure S4. For both models, we observed good recovery of the latent ability parameter (Model 5: $r = 0.873$; Model 6: $r = 0.873$). Furthermore, we observed excellent recovery for the item difficulty parameters (Model 5: $r = 0.962$; Model 6: $r = 0.962$). In contrast, we observed only adequate recovery of the item discrimination parameters (Model 5: $r = 0.667$; Model 6: $r = 0.671$). This result is not altogether unexpected given recent research that recommended at least $N = 100$ observations per item for accurate item parameter estimation (König, Spoden, & Frey, 2020). This result, however, must be interpreted in context. We demonstrate below that the less-than-ideal recovery of item discrimination parameters does not seriously impact the outputs of optimal test assembly.

Next we calculated the statistical power (i.e. true positive rate) for detecting an association between item attributes and item parameters. We defined a true positive as an association between an item attribute and parameter whose 95% highest density interval excluded zero. The results are summarized in the second column of Figure S4. For the item difficulty parameters, we detected the large association (20% variance explained) and small association (10% variance explained) in all 100 simulations. This result indicates that we were suitably powered to detect associations between item attributes and item difficulty of the magnitude reported in the manuscript. In contrast, true positive rates for contrasts involving item discrimination were smaller. For large

effects (14% variance explained), the TPR was approximately 70% (Model 5: TPR = 0.720; Model 6: TPR = 0.700). For small effects (7% variance explained), the TPR was above 40% (Model 5: TPR = 0.435; Model 6: TPR = 0.400). Thus, while our study design was moderately powered for detecting larger associations between item attributes and item discrimination, we are underpowedered for detecting smaller associations. As such, our mostly null findings for associations between item attributes and item discrimination should be interpreted with caution. (That said, if any associations do exist, they are likely small in magnitude.) Regardless, this result does not alter one of the main findings of the item calibration study; namely, item clones systematically differ in their difficulty by distractor type and are not therefore exchangeable.

Finally, we investigated the effects of item parameter misestimation on test assembly. For each simulated dataset, we submitted the ground-truth (simulated) and model-predicted (recovered) item parameters to test assembly under the same constraints used to generate the MaRs-IB short form measures. We then calculated for each test form its test information function (TIF) and IRT test score reliability (Kim & Feldt, 2010; Nicewander, 2018). Crucially, we calculated the TIF and reliability for the short forms using the true (i.e. not recovered) item parameter estimates; therefore, we can quantify how much the psychometric properties of test forms are impacted by suboptimal item selection due to parameter estimation noise. The results are summarized in the third and fourth columns of Figure S4. As is expected, TIF was larger for the test forms made using the true item parameters, indicating that estimation noise can lead to suboptimal item selection. Despite this, estimation noise led to only marginal decrements in IRT test reliability. For item parameters estimated using Model 5, parameter noise resulted in an average loss of score reliability of $\Delta\rho = 0.020$ (SD = 0.014). For item parameters estimated using Model 6, parameter noise resulted in an average loss of score reliability of $\Delta\rho = 0.019$ (SD = 0.013).

In sum, our sample size and experiment design was sufficient with regard to our study objectives. The results of the parameter recovery analysis demonstrate that we are able to estimate item difficulty parameters, and their association with item

attributes, with excellent precision. In contrast, we were able to estimate item discrimination parameters, and their association with item attributes, only with moderate precision. Because the observed variability in item discrimination parameters were small, however, imprecision in the estimation of these parameters yields minimal impact on subsequent test assembly. Indeed, the loss of score reliability due to suboptimal item selection was negligible.

References

Carpenter, B., Gelman, A., Hoffman, M. D., Lee, D., Goodrich, B., Betancourt, M., . . . Riddell, A. (2017). Stan: A probabilistic programming language. *Journal of statistical software*, *76*(1), 1–32.

Chierchia, G., Fuhrmann, D., Knoll, L. J., Pi-Sunyer, B. P., Sakhardande, A. L., & Blakemore, S.-J. (2019). The matrix reasoning item bank (MaRs-IB): novel, open-access abstract reasoning items for adolescents and adults. *Royal Society open science*, *6*(10), 190232.

Chiesi, F., Morsanyi, K., Donati, M. A., & Primi, C. (2018). Applying item response theory to develop a shortened version of the need for cognition scale. *Advances in cognitive psychology*, *14*(3), 75.

Fagerlin, A., Zikmund-Fisher, B. J., Ubel, P. A., Jankovic, A., Derry, H. A., & Smith, D. M. (2007). Measuring numeracy without a math test: development of the subjective numeracy scale. *Medical Decision Making*, *27*(5), 672–680.

Iverson, G. L., Marsh, J. M., Connors, E. J., & Terry, D. P. (2021). Normative reference values, reliability, and item-level symptom endorsement for the PROMIS® v2. 0 cognitive function-short forms 4a, 6a and 8a. *Archives of Clinical Neuropsychology*.

Kim, S., & Feldt, L. S. (2010). The estimation of the irt reliability coefficient and its lower and upper bounds, with comparisons to ctt reliability statistics. *Asia Pacific Education Review*, *11*(2), 179–188.

König, C., Spoden, C., & Frey, A. (2020). An optimized bayesian hierarchical two-parameter logistic model for small-sample item calibration. *Applied psychological measurement*, *44*(4), 311–326.

Nicewander, W. A. (2018). Conditional reliability coefficients for test scores. *Psychological Methods*, *23*(2), 351.

Wise, S. L. (2017). Rapid-guessing behavior: Its identification, interpretation, and implications. *Educational Measurement: Issues and Practice*, *36*(4), 52–61.

Wise, S. L., & DeMars, C. E. (2006). An application of item response time: The

effort-moderated irt model. *Journal of Educational Measurement*, *43*(1), 19–38.

Model	psis-loco	Δ psis-loco (se)
1r	28376.7	635.3 (29.7)
1	27741.4	-
2r	27627.6	665.6 (32.2)
2	26962.1	-
3r	27218.7	633.1 (33.9)
3	26585.5	-

Table S1

Comparison of the first wave of item response models to their equivalents without a guessing parameter ($\gamma = 0$). LOO-CV values are presented in deviance scale (i.e. smaller values indicate better fit). Abbreviations: PSIS = Pareto-smoothed importance sampling; LOCO = leave-one-cluster-out.

Measure	Mean (SD)	IQR	Spearman rank correlation		
			NFC-10	PCF-8a	SNS
NFC-10	24.44 (9.01)	19 – 31	-		
PCF-8a	22.58 (6.45)	18 – 28	0.28**	-	
SNS	29.02 (7.36)	25 – 35	0.42**	0.32**	-
MaRs-IB SF	8.00 (2.53)	6 – 10	-0.04	0.04	0.14*
MaRs-IB LF	15.73 (5.13)	12 – 20	0.12*	-0.06	0.18*

Table S2

Correlations between performance on the MaRs-IB short/long form and self-report measures. Abbreviations: IQR = interquartile range; NFC-10 = need for cognition (10-item) scale (Chiesi, Morsanyi, Donati, & Primi, 2018); PCF = PROMIS cognitive functioning scale (8a; Iverson, Marsh, Connors, & Terry 2021); SNS = subjective numeracy scale (Fagerlin et al., 2007). ** $p < 0.001$, * $p < 0.05$ (not corrected for multiple comparisons)

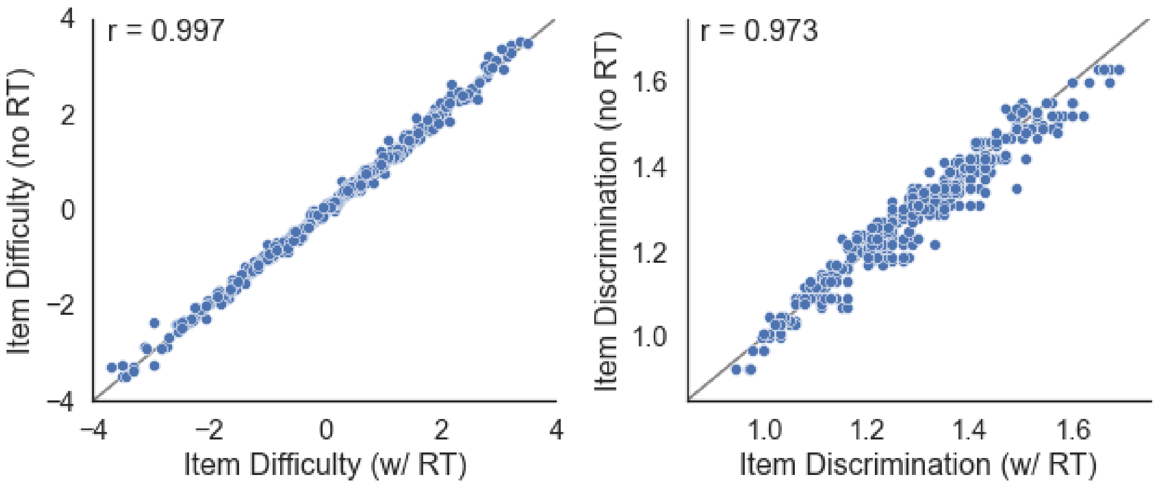

Figure S1. Item parameter estimates for the best-fitting model (Model 5) with and without including mean response time as a clone-level attribute.

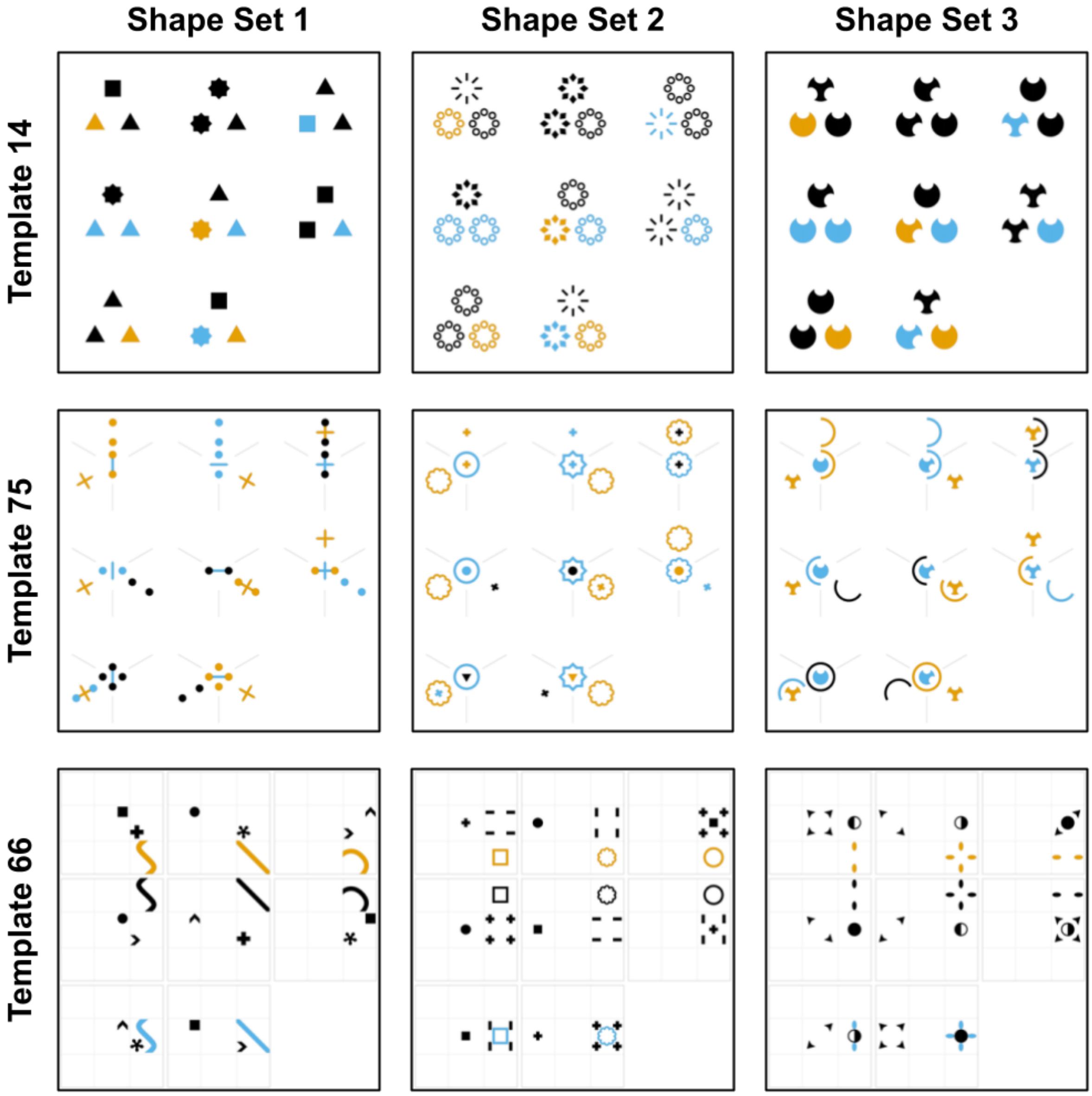

Figure S2. An example item clone from each of the nine stimulus sets.

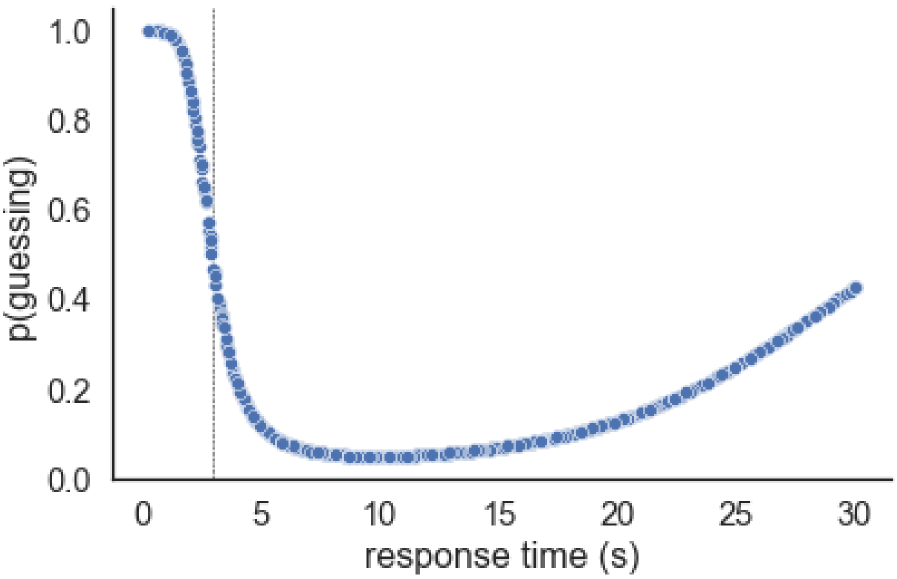

Figure S3. Estimates of the rapid guessing weights (w_{ij}) and their corresponding responses times from the effort-moderated item response theory (EM-IRT) model fit to response data from N=180 pilot participants. The dashed line indicates the chosen rapid guessing threshold, i.e. where $w = 0.5$.

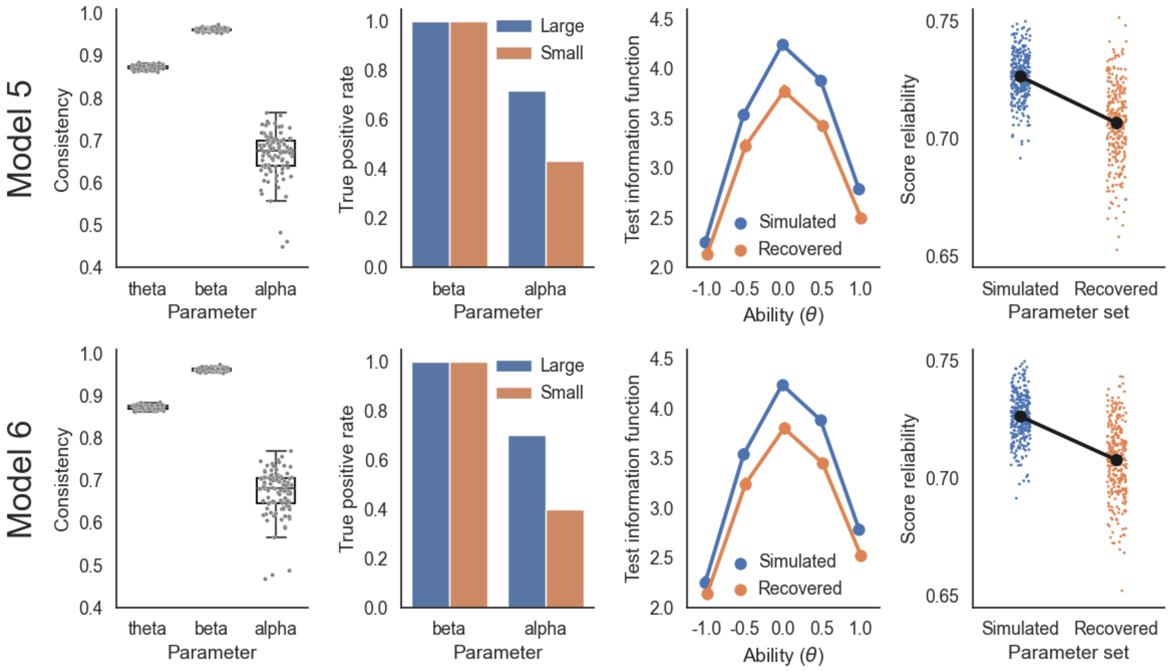

Figure S4. Summary of results from the parameter recovery & power analysis. (Column 1) Pearson correlation coefficients between the ground-truth and recovered model parameters. (Column 2) True positive rates for detecting an association between item attributes and item parameters. For the item difficulty parameters (beta), large and small effects indicate 20% and 10% variance explained, respectively. For the item discrimination parameters (alpha), large and small effects indicate 14% and 7% variance explained, respectively. (Column 3) Test information function estimates for the test forms produced using the ground-truth (simulated; blue) and recovered (orange) item parameter estimates. (Column 4) IRT test score reliability estimates for the test forms produced using the ground-truth (simulated; blue) and recovered (orange) item parameter estimates.
